# Ecological-network models link diversity, structure and function in the plankton food-web

**DOI:** 10.1038/srep21806

**Published:** 2016-02-17

**Authors:** Domenico D’Alelio, Simone Libralato, Timothy Wyatt, Maurizio Ribera d’Alcalà

**Affiliations:** 1 Stazione Zoologica Anton Dohrn, Department of Integrative Marine Ecology, Villa Comunale, 80121, Napoli, Italy; 2 OGS (Istituto Nazionale di Oceanografia e di Geofisica Sperimentale), Oceanography Division, Via Beirut 2/4 (Ex-Sissa building), 34151, Trieste, Italy; 3 Spanish National Research Council, Instituto de Investigaciones Marinas de Vigo, Vigo, Spain

## Abstract

A planktonic food-web model including sixty-three functional nodes (representing auto- mixo- and heterotrophs) was developed to integrate most trophic diversity present in the plankton. The model was implemented in two variants - which we named ‘green’ and ‘blue’ - characterized by opposite amounts of phytoplankton biomass and representing, respectively, bloom and non-bloom states of the system. Taxonomically disaggregated food-webs described herein allowed to shed light on how components of the plankton community changed their trophic behavior in the two different conditions, and modified the overall functioning of the plankton food web. The green and blue food-webs showed distinct organizations in terms of trophic roles of the nodes and carbon fluxes between them. Such re-organization stemmed from switches in selective grazing by both metazoan and protozoan consumers. Switches in food-web structure resulted in relatively small differences in the efficiency of material transfer towards higher trophic levels. For instance, from green to blue states, a seven-fold decrease in phytoplankton biomass translated into only a two-fold decrease in potential planktivorous fish biomass. By linking diversity, structure and function in the plankton food-web, we discuss the role of internal mechanisms, relying on species-specific functionalities, in driving the ‘adaptive’ responses of plankton communities to perturbations.

The evolutionary causes and ecological implications of plankton diversity have challenged ecologists ever since Hutchinson’s classical paper^1^. He wrote: “…how it is possible for a number of species to coexist in a relatively isotropic or unstructured environment all competing for the same sorts of materials?”. Biological diversity and community organization are believed to have a central role in the functioning of ecosystems in general^2^,^3^,^4^. However, in most holistic approaches to aquatic systems, plankton diversity and food-web structure are rarely detailed^5^,^6^. In contrast, detailed ‘food-web’ studies, i.e., those focusing on species-species interactions, trophic links and cascading effects, are considered as the most appropriate approaches to integrate biodiversity, structure, i.e. community organization, and function, i.e. energy and elemental fluxes^7^,^8^.

Plankton is very diverse taxonomically, and encompasses extremely distant groups in evolutionary terms^9^. It includes unicellular and multicellular, autotrophic and heterotrophic organisms^9^, occurring in highly-timed populations’ successions^10^,^11^,^12^, organized in complex communities^13^,^14^ and deeply entangled in food-webs and biogeochemical cycles^15^,^16^,^17^. But little attention has been paid to the profusion of trophic processes among plankton organisms (i.e., the real plankton food-web)^18^, either by biogeochemical or fishery modelling, based on either the nutrient-phytoplankton-zooplankton scheme (NPZ^19^) or the slightly more detailed ‘plankton functional type’ (e.g., as in the BFM approach, http://bfm-community.eu) approaches. Both approaches aggregate plankton organisms into a few large groups ruled by two or a few trophic steps, and thus ignore a large number of species and trophic processes. On the other hand, the more recent and promising ‘trait-based’ approach, which considers morphological, functional and behavioral diversity in full, is still in its infancy^20^.

The trophic diversity of plankton is huge. For instance, planktonic protists can eat other unicellular organisms^21^,^22^ and close the very first step of the pelagic food-chain within ‘trophic loops’^23^,^24^. Moreover, mesozooplankton (i.e. metazoans) include filter-feeding animals^25^, suspension feeders capable of both prey-selectivity and omnivory^26^,^27^, obligate and occasional predators (i.e., eat other metazoans)^26^,^28^. All this diversity, in both components and interactions, should be integrated in realistic models, either conceptual or numerical, to better reconstruct plankton functioning in time and space. These models should be ‘network models’^29^,^30^, which considerably zoom in the hierarchical organization of the basal portion of aquatic food-webs and incorporate biological detail in species-interactions, so that the roles played by individual species in ecosystem function can be explored^31^.

This study is an attempt to move in that direction. Here we describe the roles of species and trophic diversity in the functioning of a plankton community (e.g., amounts and pathways of biomass transfer) by analyzing a plankton food-web in two states, at relatively high and low biomass levels. We constructed a food-web model (FWM) to simulate a highly resolved plankton community, which includes sixty-three linked functional nodes (FN). This number of FNs greatly exceeds the minimum resolution considered sufficient for ecological network studies^32^, but enables us to represent most of the trophic diversity in the plankton community.

Our plankton food-web was modeled according to ecological network methodologies – in which trophic links are weighted by material fluxes^33^ – as in the ‘Ecopath’ approach (http://www.ecopath.org/)^34^ – , often used to derive biomass fluxes in exploited food-webs^35^. We discuss the role of internal mechanisms, relying on species-specific functionalities, which enable the ‘adaptive’ responses of plankton communities to changes in trophic regime. Our results reinforce the view that detailed studies of food-web structures and functioning are necessary to understand the link between biodiversity and functioning of plankton communities.

## Conceptual structure and setup of the plankton FWM

We studied the plankton community monitored at the Long-Term-Ecological Research station MareChiara in the Gulf of Naples (LTER-MC in GoN, depth = −75 m^36^) during summer. At this coastal site, bloom and non-bloom (herein named ‘green’ and ‘blue’) states alternated during most of the Mediterranean summer (mid-June-late August, years 2002–2009)^37^. The summer LTER-MC plankton series has already been studied with a simpler but informative food-web approach in a previous study, where possible biological links were inferred from positive and negative co-variations in abundance of the FNs of the plankton food-web (both unicellular and multicellular organisms) for the two states^37^. There, the changes in links that marked transitions between states reflected the switching of grazing pressure from one set of prey to another, generally due to changes in the quantity and quality of microbial biomass^37^. This trophic flexibility was named ‘community plasticity’. Here, we will test the robustness of that plasticity in a quantitative way. The plankton FWM has been implemented based on the following five conceptual and numerical steps (details concerning the steps for the web construction and state-of-the-art information on plankton can be found in the Supplementary Information (SI); raw input and output data of the FWM are shown in Supplementary Data 1–5).

(1) The computational method is based on the conceptual and numerical approach of Ecopath. This assumes mass-balance (i.e. consumption equals outflows in terms of production, respiration and unassimilated food for each FN) and computes biomass flows between nodes^34^ (see M&M and SI for the derivation of physiological rates).

(2) The Carbon-based biomasses of the FNs (Table 1, see also M&M and SI for a description of their derivation) are tightly linked to *in situ* observations. But some subjective decisions, based on expert knowledge and literature information, have been taken. These are: i) the initial ranges assumed by physiological rates of the FNs; ii) the trophic links among FNs; iii) the weights of these links.

(3) The partially subjective construction of the web is tested by generating alternative network structures (different values of trophic parameters and link-weights) by means of a Monte Carlo Markov Chain (MCMC) method (see M&M). The best models for the green and blue states are chosen with the constraints that the overall amount of biomass production, dissipation and transfer across the web of nodes must: i) conserve the biomass at each node; ii) guarantee the whole system metabolism, *sensu lato*, i.e. the balance between the sums of FNs’ production and consumption; iii) guarantee for each FN the balance between production and consumption; iv) determine efficiency of energy transfer from a prey to a predator <1; v) determine respiration for consumers >0. Moreover, at each MCMC step, very weak links were neglected (i.e. when a prey contributed to less than 0.01% to predator’s diet). For both states, few solutions lead the system to biomass-balance, and balanced green and blue models are both characterized by primary production rates at the upper boundary and consumption rates at the lower boundary of the initial ranges.

(4) We locate the synthetic food-web spatially in a 60-meter water column, where most trophic processes in the plankton occur in the GoN, due to low-light conditions and sediment re-suspension below that depth^36^. Unicellular organisms are assigned to either the surface or the subsurface layers (0 to −5 m and −5 to −60 m, respectively), which are separated by a steep density-gradient in the GoN during summer^36^. Mesozooplankton and *Myrionecta rubra* are not separated between layers, since they can perform large-scale diel vertical migrations across the pycnocline^38^,^39^. Thus mesozooplankton in the model can feed in both layers (see SI for more detail).

(5) To better characterize the structure of the network and the roles of nodes, we use different diagnostic food-web indicators. Four indicators describe the role each node can play in system function. These are: ‘weighted degree’, i.e. the rank of FNs based on the amount of biomass taken from and delivered to other FNs (in this context, we consider main ‘hubs’ the first five nodes in the weight degree ranking); ‘trophic level’ (TL) of each FN based on the average number of trophic steps from TL = 1 (the primary producers) to the same FN; ‘keystoneness’, i.e. a measure to highlight keystone FNs, or those which, despite having low biomass, can induce large changes in biomass of other FNs^40^; ‘overall relative effect’, i.e. a measure of the impact that a small change in the biomass of a node has on the biomass of all other nodes of the food web^41^; the contribution of each FN to ‘relative ascendency’, i.e. the mutual information in a system scaled by system throughput^42^, that measures the degree of organization of a system and its capability to cope with perturbations^43^. Keystoneness and overall relative effect are derived from ‘mixed trophic impact’ matrices, produced according to Libralato *et al*.^41^ and shown in Supplementary Data 5. Another descriptor results from lumping biomasses and flows. This is the ‘transfer efficiency’ (TE), i.e., the percentage of net production at trophic level *n* that is converted into production by level *n+1*. In practice, flows and biomass are aggregated in simplified grazing and detritus chains called ‘Lindeman spines’ (Ls^44^) as modified by Libralato *et al*.^45^. Ls are homologous to the original complex web of flows, but permit easier analyses of biomass-transfers from lower to higher trophic levels. We also estimate the number and length of ‘predatory cycles’, i.e. pathways that start and end at the same FN, with the exclusion of any non-living node or detrital form.

## Results

### Switching roles, changing flows

**Figure 1:**
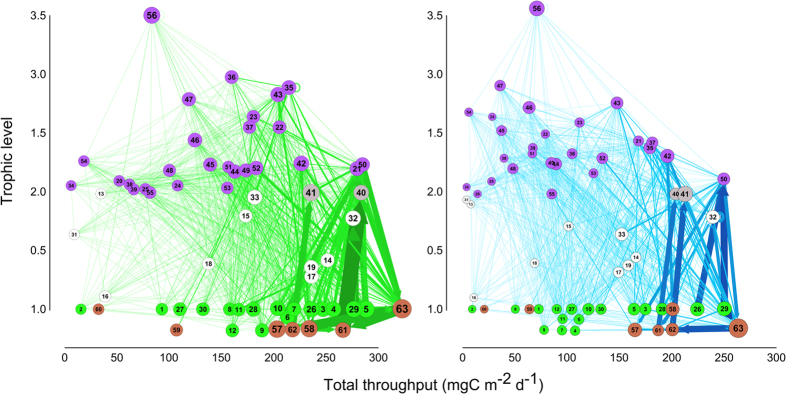
Green and blue plankton food webs. Node colors distinguish non living (brown), primary producers (green), mixotrophic groups (light grey) and consumers (purple). Node size is proportional to log biomass and edge size is proportional to flows. Groups are displaced in xy space by TL (y-axis) and log total throughput of the node (x-axis). Some groups, conventionally at TL = 1 are displaced at lower TL for clarity.

The plankton community in the GoN was characterized by a different amount of biomass in the two states (Table 1). Surface layers showed relatively higher concentrations of unicellular organisms than the deeper layers, in both green and blue states, but the green surface layer was seven-fold richer in phytoplankton than the blue one. Diatoms dominated phytoplankton in the green surface layer. Yet, mesozooplankton biomass was somewhat identical between the two states. Overall, a slightly higher diversity was present in the green state of the system (i.e. Shannon’s H accounted for 3.2 vs. 2.8). Despite the fact that green and blue food-webs were built starting from the same diet-matrix (the plankton community was the same in the green and blue states) and the majority of trophic links were present in both webs (>95%; Supplementary Fig. S1; Supplementary Data 5), the final Carbon-fluxes-matrices deriving from MCMC computations were similar but not identical (Mantel test: R = 0.79; p < 0.001; Supplementary Data 4–5) and the same trophic links showed distinct biomass-flow intensities between the green and blue states (Fig. 1). The alternative patterns in biomass flows resulted from the physiological and trophic adjustments in the FNs in response to biomass differences in the two states. For instance, many FNs (e.g., #24, 35, 36, 45, 54) changed their topological position in the web, suggesting a swing in the role played in driving biomass flows. When passing from green to blue states, the food web underwent the following three main trophic modifications.

**Figure 2:**
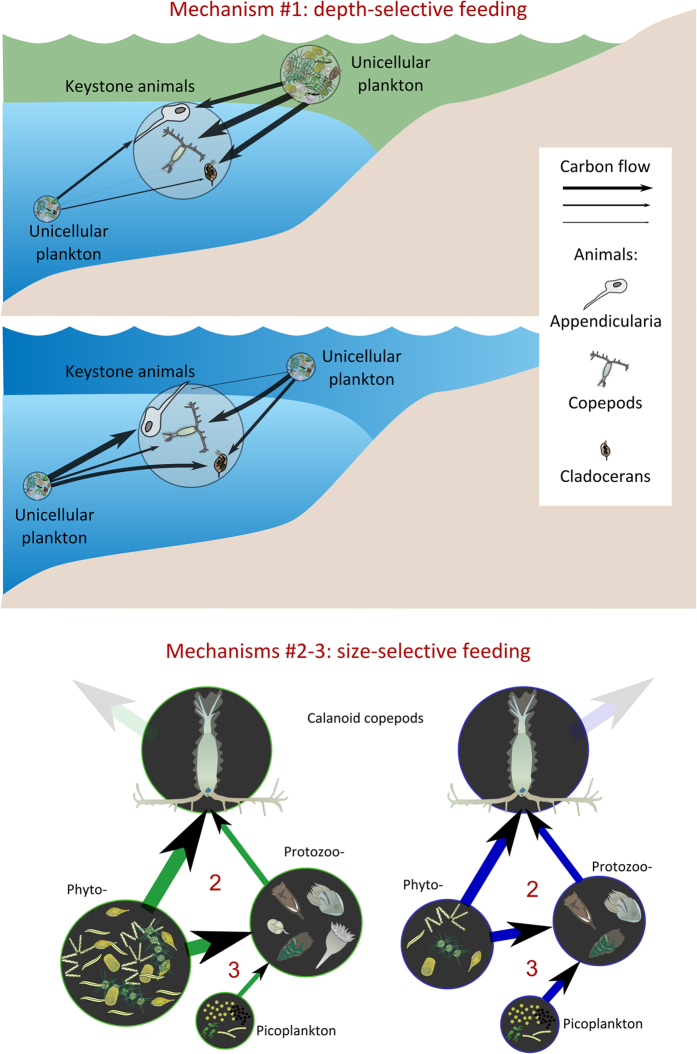
Schematic depiction of the main differences in trophic fluxes among the green and blue states. In the uppermost panels, the increasing of material fluxes from the deeper layer to the main metazoan consumers. In the lowermost schemes, from green to blue states, the increasing of fluxes from picoplankton to protozoan consumers and from the latter to calanoid copepods.

**Figure 3:**
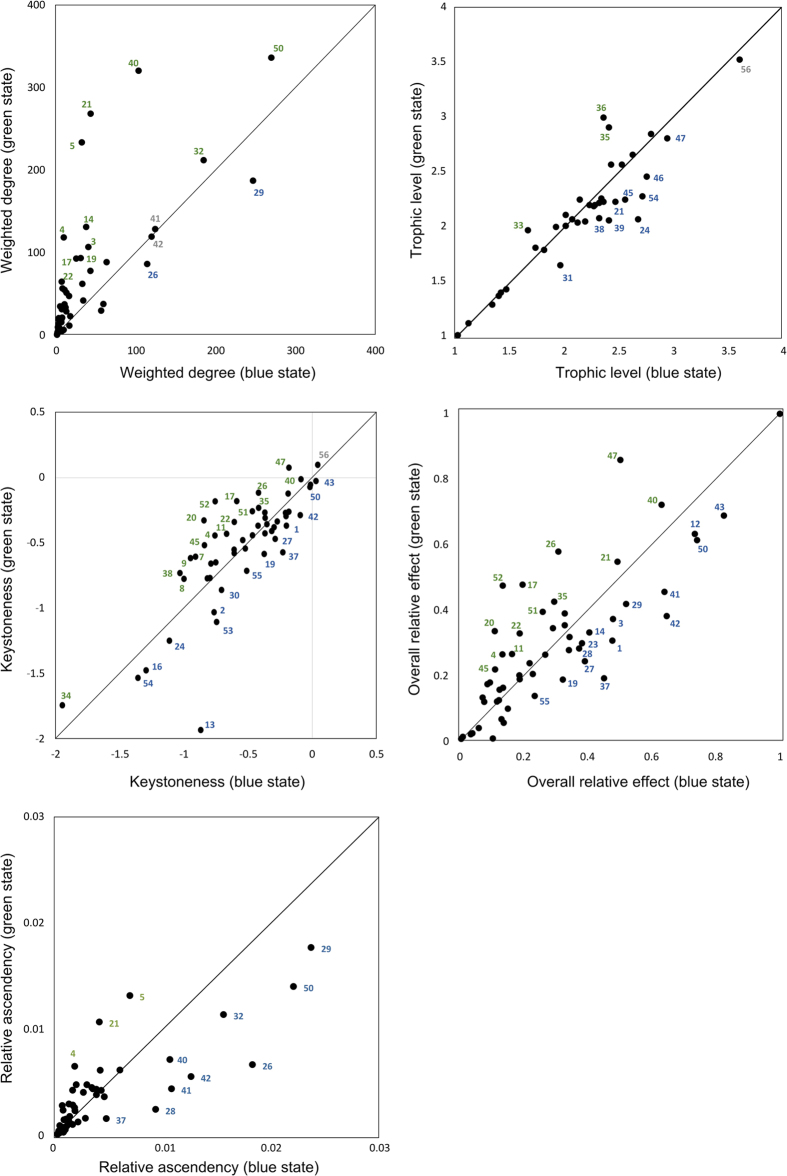
Comparison between food-web indicators in the green and blue webs (on the y and x axes, respectively). Numbers refer to FNs’ codes. Those FNs showing a relatively higher value for a food-web indicator in one of the two webs are either green- or blue-colored.

(1) Mesozooplankton fed preferentially in the deeper layer in the blue than in the green state (Fig. 2; Supplementary Fig. S2). This feature, although common to most mesozooplankton groups in our model, was clearly detectable in the main players, which were (according to analyses shown in Fig. 3): the filter-feeding Appendicularia (FN 50, energy hub in both states, which exerted a high impact in both webs), the cladoceran *Penilia avirostris* (FN 42, an energy hub at both states, with a moderate impact on the blue web) and the calanoid copepod *Centropages typicus* (FN 47, not an energy hub but which has a high impact on the green web). Their consumption of unicellular plankton in the deeper layer changed from 37, 17 and 1% to 91, 55 and 32% of the total biomass, respectively, between the green and blue state (Supplementary Data 4).

(2) Calanoid copepods (FN44-49), dominant in terms of biomass over the mesozooplankton, and main deliverer of carbon to carnivores and higher predators (e.g. fish), ate more protozooplankton in the blue state than the green (Fig. 2; Supplementary Data 4). In our FWM, protozooplankton (FN 13–25 and 31–39) contributed 33% of the diet of calanoid copepods’ during the green state (with food concentrations from >50 μg up to 250 μg C L^−1^, in the surface layer). During the blue state (food concentration was <50 μg C L^−1^ over the whole water column), that contribution rose to 41%. These estimates are in line with those from meta-analyses of experimental data^46^,^47^. Trophic flexibility is thought to make calanoid copepods resilient to phytoplankton biomass oscillations in the GoN^12^,^48^. Our FWM confirms this quantitatively, shedding light on the role played by protozooplankton in accumulating biomass from primary producers towards copepods during low-primary-production phases.

(3) Protozooplankton was deeply integrated in both green and blue webs and fed on smaller microbes in the blue than in the green state. Several mixo-/heterotrophic protists had distinct food-web indicator values between the two states (Fig. 3), suggesting that they played different roles in the two webs, i.e., as massive consumers of phytoplankton in blue phase and more autotrophic in green phase. For instance, mixotrophic nanoflagellates (FN 13) showed a remarkable increase in keystoneness while passing from the green to the blue state. This observation matches the suggestion that oligotrophic systems would be consistently driven by mixotrophic organisms^49^. In addition, many unicellular nodes had higher relative ascendency in the blue state (Fig. 3), suggesting that they played a major role in keeping system functioning at a low biomass state. In terms of material fluxes, in the more productive surface layer during the green state, the modeled protozooplankton consumed about 50% of the diatom production and 55% of total primary production, in line with empirical evidence gained for the global ocean^50^. In our model food-webs, the main protozooplankton players (*Tontonia*, *Strombidium*, heterotrophic dinoflagellates and prostomatids, in the surface layer - FNs 17,19, 21 and 22, respectively - and heterotrophic dinoflagellates and *Strobilidium* in the deeper layer - FNs 35 and 37, respectively) (Fig. 3) increased their predation rates on microbes with sizes <2 μm from 25 to 45% in the surface water layer and from 18 to 43% in the deeper layer, during the blue state (Fig. 2; Supplementary Data 4). Overall, by consuming small food particles (such as bacteria and small-sized protists) not directly edible by copepods for their limited size, mixo- and heterotrophic protozooplankton established an important pathway of delivery of organic material to metazoan consumers during the low-biomass system-state.

To synthesize, the comparison between green and blue states shows that the switch between them also changes the center of mass of fluxes from the surface to the subsurface layers. While the green state was mainly driven by diatom biomass - especially *Leptocylindrus* spp. (FN 5) - from the surface layer, in the blue state, organic material passed more intensely from smaller microbes in the sub-surface layer to filter-feeding animals, like Appendicularia, eating mainly pico and nanoplankton^51^. Non-filter feeding animals, like copepods, overcame low-phytoplankton biomass states by eating protozooplankton. As both predators and prey, protozooplankton appeared to buffer biomass-flows in the web during the shifts in system’s productivity, acting either as massive consumers of phytoplankton during bloom conditions or as accumulators of biomass during non-bloom phases.

### Food-web changes and system functioning

Despite having the same *vertical* extension (i.e. from plankton producers to carnivores at maximum TL ~ 3.5 in both states) (Fig. 1), the food-web underwent important modifications in its structure during switches between the two system-states (Figs 1, 2, 3 and former section), which affected system functionality.

**Figure 4:**
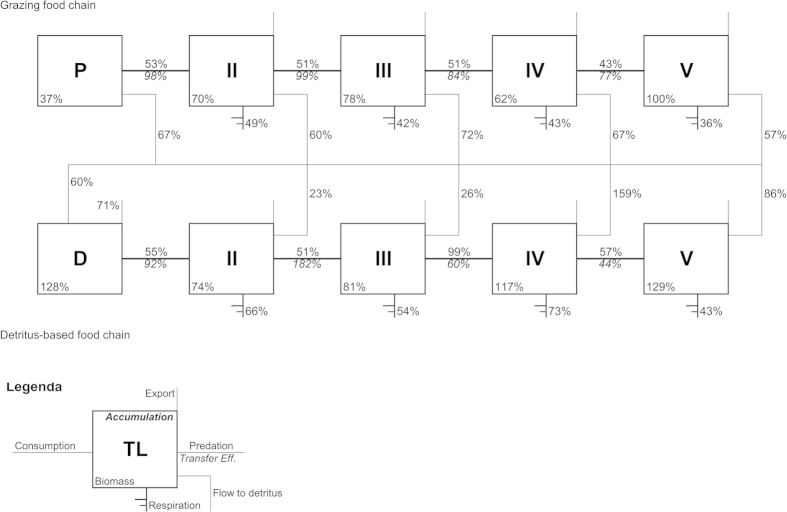
Lindeman spine. Grazing food chain (upper part) and detritus-based food chain (lower chain). Ratio between values referred to blue and green states are shown as percent.

**Figure 5:**
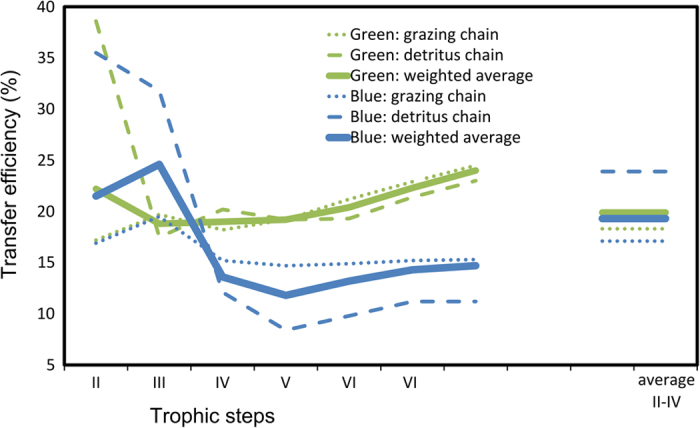
Transfer efficiencies for each trophic step (II = step from TL1 to TL 2). Weighted averages are conventionally reported for the first 4 TLs.

In the plankton system modeled here, the major part of organic material entering the system from primary production was dissipated at the lower levels of the food-web. Part of the production was recycled through non-living organic forms (detritus), thus fueling the detritus-based food chain that had lower but comparable intensity to the grazing chain (Fig. 4). In spite of large differences in system flows of carbon (during the blue state, they were approximately 50% less than during the green state), the plankton food-web tended to maintain its overall TE around ~20% in both states (Fig. 5), corroborating the observation that TE is unrelated to system productivity in marine plankton^52^.

A 20% average TE contrasts with the usual 10% value found by large meta-analyses of marine ecosystem models representing from plankton to fish^53^ (which confirmed previous general expectations^54^) and with the 12–14% TE values derived in more recent studies on continental shelves^55^. In our FWM, the overall TE for detritus-based food-chains was similar in blue and green states, suggesting that detritus is also an important driver of the system. However, TE was slightly higher for the grazing food-chain during the green state (18.3 vs. 17.1%). Overall, the high values of TE found here are in the upper part of the range found in meta-analyses, thus are not incompatible with general values and whole ecosystem estimates^56^. The result is important because it emphasizes the higher importance of planktonic trophic interactions, which usually dominate lower TLs of marine webs.

The trend of TE across trophic steps (from TL to TL) provided us with important information on system functioning (Fig. 5). Concerning the grazing chain, at trophic step II (i.e., from TL1 to TL2, mainly herbivorous protists), TE was above 20% during both system states, as observed in the Baltic Sea and freshwater lakes for a phytoplankton-dominated food-web, but in which grazing of protozoa on microalgae was not considered^57^,^58^. In addition, in both blue and green states, an even higher efficiency (35.5 and 38.6%) was found at trophic step II of the detritus chain, due to bacterial activity and consequent transfer of material from bacteria to protozooplankton.

Between trophic step II and III in the two system states, TE was rising in blue and decreasing in green (Fig. 5). This is related to a higher intra-guild predation in protozooplankton (and thus energy dissipation) during the green state (as suggested by a previous study^59^), supported in our web by the presence of longer predatory cycles (i.e. cycles with a higher number of trophic steps), in comparison with the blue state (Supplementary Fig. S3). Between trophic steps II and III, the higher TE in blue state contrasts the low productivity of the system and, conversely, the lower TE in green represents a higher dissipation of energy in case of high production. Furthermore, dissipation is higher (efficiency is lower) in blue state at trophic steps higher than III. The following trophic steps (IV, V, etc.) highlighted the large availability of production for successive TLs in green state; conversely, in blue state, where metazoans composing these TLs had similar biomass as in green (Table 1), a consistent part of energy was needed to maintain such biomass (through respiration and minimum natural mortality) thus reducing the TE (Fig. 5).

Although higher planktonic TLs contribute less to the energy flow, any large difference in TE at those stages of the web is relevant for predators with higher TL (such as fish)^53^. These results translate into a production by consumers unused in the plankton food web equal to 216.7 mgC m^−2^ day^−1^ and 421.5 mgC m^−2^ day^−1^ that are hypothetically available at higher TLs during blue and green conditions, respectively. By neglecting any natural mortality for plankton consumers and by considering an average TE of 10% for higher trophic levels and the ratio g of C/g of dry weight = 1/9, the above figures correspond to the daily needs for 0.19 and 0.38 ton per km^2^ of planktivorous fish, respectively, in the blue and green states. Although there are no assessments available in the studied area, these values are compatible with results for the northern part of the Tyrrhenian Sea^60^. Remarkably, a seven-fold increase in phytoplankton biomass, based on our calculation, would translate into a only ~2-fold increase in fish biomass, due to energy dissipation needed to sustain plankton functioning.

According to recent analysis of predator-prey biomass in both terrestrial and aquatic ecosystems, a remarkable portion of prey biomass is not transferred to the upper trophic level under higher prey abundance, resulting in a biomass pyramid that is increasingly bottom heavy at higher biomass^4^. The ecological mechanism behind such regular organization of ecosystems on Earth has been only partially explained by the density-dependence dynamics of feeding processes^4^. Although our analysis (see Lindeman Spine in Fig. 4) confirms the tendency to have bottom-heavier distribution of biomass in the more productive situation (green state), this pattern is dissimilar from the ‘classic’ pyramid. More specifically, the distribution of biomass across trophic levels showed that intermediate trophic levels assume a relatively higher weight in terms of material retention (see Lindeman Spine in Fig. 4), thus displaying a sort of rhomboid already found as typical of marine ecosystems^61^. Based on the observations that a large fraction of organic matter in plankton food-webs is consumed and recycled at the level of unicellular consumers, we suggest that rhomboid TL-biomass distributions in pelagic systems would emerge due to the activity of protozooplankton grazing, which limits the transfer of biomass to metazoan zooplankton but, on the other hand, provides the system with the ability to counteract strong oscillations in primary production.

## Discussion

We constructed a food-web model for a plankton community in the coastal Gulf of Naples during summer based on *in situ* data and literature information. The components were included with unprecedented taxonomic detail and up-to-date estimates of physiological parameters. Our dataset does not include macroplankton, whose presence, even considering the bias of the sampling technique, appeared as not significant at the site during the periods analyzed in this study.

The web was tested for two different trophic conditions, named green and blue states, corresponding to relatively high- and low-phytoplankton biomass observed *in situ*. Although all node-biomasses were different between the two states, these were mainly differentiated by the contribution of diatoms to total phytoplankton biomass. The modeled community persisted for several weeks in summer and recurred over the eight years that were analyzed^36^,^37^. The steady state assumption for green and blue states, used to apply the mass balance procedure of Ecopath, is thus reasonable in view of the duration of each of the two states for more than a week^36^,^37^ and the high turnover rates of plankton groups.

The high taxonomic resolution introduced in our model enabled us to link the pattern of biomass-fluxes to food-web modifications and to show that key components of the web modify their relevance during the two states. Nonetheless, aggregation and resolution settings are highly problem-specific. On the one hand, the gross aggregation of plankton has been efficiently used for studying ecosystem properties^35^,^56^ and for linking plankton dynamics with main biogeochemical processes^19^,^62^. On the other hand, the increase of functional groups and the definition of precise trophic links among them can shed significant light on the mechanisms behind ecosystem dynamics (e.g., Boit and co-authors^6^). Furthermore, given the increasing information on selective predation by metazoan consumers, the detailed plankton networks might help better explaining some dynamics of planktivorous fish^63^.

In the specific case we analyzed, the highest TL was 3.5 for both eutrophic and oligotrophic states and there were material flows that were transferring energy over more than 5 passages (although fluxes were minimal in upper passages), suggesting that, even in plankton the high number of trophic interactions permits the emergence of a complex trophic structure^64^. Highly remarkable is the asymmetry between carbon-flow at the level of bacteria and protists and that at the level of small metazoans, with the latter being towards low values of carbon uptake despite co-occurring high values in primary production. This translates into dissipation at the lower levels, with recycling within the first two TLs proportional to production. But material flow beyond plankton would still co-vary with primary production, although not linearly. This ‘inefficiency’ in transfer is partially compensated by the fact that the predators of the plankton community during the oligotrophic/blue state are limited not only by the low production but also by the lower TE.

One may wonder why the plankton system should dissipate energy to such a high extent, since, even with a lower flux of material, there was no loss of nodes and most of the links were maintained. Our hypothesis is that energy dissipation is the mechanism by which the plasticity of the web, which resides in its diversity, is preserved. As already suggested for both aquatic and terrestrial ecosystems, biological diversity and trophic multifunctionality are strongly linked, especially if diversity is high at the level of primary consumers^65^. In our FWM, the increased biomass of primary producers in the green compared with the blue state, besides increasing the consumption of organic material at the lower trophic levels, is followed by an increase of predatory cycles (Supplementary Fig. S3), i.e. circular trophic paths potentially stabilizing food-webs (Thompson and co-authors^8^ and reference therein), which include many mixo- and heterotrophic protists. In our FWM, by promoting multiple trophic paths, diversity buffers the overall flux upward in the web and reduces flux-variability. The carbon of these blooms which is not exported to deeper layers or to herbivorous fish larvae is re-cycled with high efficiency within the first two trophic levels, as defined in this study. This results in higher retention of the elemental pool in the surface layer, efficient recycling to fuel primary production and higher diversity. The latter, in turn, is favored by frequent transitions from one steady state to another which imply, as shown, the changes in the roles of each species in the web. On the other hand, diversity reinforces itself by keeping the resistance of community against perturbations and by promoting species persistence, as suggested by theoretical modeling studies^66^.

As a consequence of higher material ‘dissipation’ at the first trophic steps of the green web, stemming from the internal recycling, the potential output of material and energy from the plankton food-web (for instance to fish) is adjusted to a low average level in both states. We can hypothesize that among the small planktonic metazoans natural selection has favored those that can grow and reproduce at lower food levels, i.e. the dominant condition in the recent ocean, and thus profit only partially from the carbon bursts of phytoplankton blooms. Such a trend would have been promoted in higher taxa too. For instance, upwelling regions (affected by strong oscillations in the available biomass at the lower level of the pelagic food-web, such as the Benguela system^67^) are alternately dominated by two kinds of planktivorous fish, with different trophic strategies^63^: while anchovies eat mainly larger zooplankton, and dominate during stronger upwelling (higher phytoplankton biomass) regimes, sardines, able to capture phytoplankton and smaller zooplankton, dominate during weaker upwelling (lower phytoplankton biomass). The evolutionary significance of this alternation would be to optimize biomass transfer under different productivity conditions based on the variety of food available.

Compared to terrestrial ecosystems, longer food-chains would arise in aquatic systems^7^,^68^,^69^. The number of trophic levels in aquatic pelagic communities of lakes and oceans is thought to be rarely less than four to five due to the presence of the microbial loop, phagotrophic algae and omnivorous zooplankton, while terrestrial systems are more constrained and seldom exceed three trophic levels^7^,^68^,^69^. A progressive-biomass-accumulation pattern was historically observed in relatively ‘simpler’ trophic chains (composed by primary producers= > herbivorous zooplankton= > carnivorous zooplankton= > planktivorous fish= > top predators, see^70^). Our observations based on highly resolved plankton food webs suggest a progressive expansion of trophic steps at the basal level of the web due to the need of progressive biomass accumulation via gradual assimilation steps in the same planktonic domain. This leads us to speculate that the plankton ‘niche’ is never empty, because of the specific characteristic of dilute aquatic environments, and also highlights why there has been such a strong convergence towards small sizes and unicellularity in plankton for dramatically distant groups, which nevertheless have similar trophic roles^9^.

These considerations imply that the trophic habits of plankton cannot become too specialized, or at least that specialized strategies are rare (for example as reported by Lombard and co-authors^71^). Plankton survival depends on the possibility to handle the variability that at sea is mainly in the concentrations and forms of substrates, besides the light. Planktonic organisms must be trophically plastic and when they are not, they must act in consortium, as suggested by^13^,^14^,^37^, which may be another reason why the system dissipates so much energy internally. The collective response of plankton to perturbation is also supported by the fact that at the lowest biomass state the web is more organized than at the highest biomass state, as supported by the observation that the overall system ascendancy was higher in blue (31.1% of the system capacity) than in green web (28.3%) (Fig. 3). We showed that the whole food-web re-organizes based on the shift in primary producers’ biomass by adopting distinct biomass-flux patterns in the blue and green states of the system: this may represent an emergent collective behavior resulting from the series of changes/effects/feedbacks among organisms, which translates into a response at the community level (‘community plasticity’) to negative and positive variations in primary production.

We conclude that:

- Disaggregated food webs shed light on how components of the plankton community change in two different trophic conditions, and modify the overall functioning of plankton food web. For instance, our FWM permits explicit and detailed representation of the hierarchy in roles of protozooplankton (one of the most neglected planktonic components) and highlights its role in guaranteeing system response to different environmental conditions^15^.
- Detailed plankton network-models provide new ways to investigate the response of plankton communities to perturbations, like coastal eutrophication^72^ and ocean warming^73^ or species invasions^74^ in a global-change context and might contribute to ecosystem health assessment. Moreover, the detailed description of plankton communities also serves as a better basis for predicting the use of the plankton production by higher trophic levels, including species exploited for human consumption.
- Plankton food-web models with better resolution of the functional components can reveal the underlying mechanisms that link diversity, biogeochemical processes and ecosystem functioning, promote concepts developed in general ecological contexts^2^,^3^,^4^,^5^, shed some light on ‘ancient’ paradoxes such as Hutchinson’s^1^, complement current interpretations of plankton succession^10^,^11^,^12^, and contribute to explanation of the high degree of species interconnection observed in plankton communities^13^,^14^.
- This study emphasized that high plankton biodiversity is matched by high functional diversity resulting in a plethora of trophic niches, despite the apparently simple pelagic environment. Ultimately, functional diversity and species-interconnections are not paradoxical but essential features needed to sustain plankton ecosystem function.

## Materials and Methods

The input data of the FWM are indicated by the following points.

(A) the summer-averaged Carbon-biomasses (Bi, as mgC m^−2^) of plankton FNs (among which, 17 for phytoplankton, 10 for mixotrophic-protozooplankton, 12 for heterotrophic-protozooplankton, 2 for heterotrophic bacteria, 5 for particulate detritus, 15 for mesozooplankton and 2 for Dissolved Organic Carbon (DOC), Table 1) were derived from LTER-MC data (as described in Supplementary Methods in SI) and corroborated by data in the literature referring to individual Carbon-content for living and non-living nodes.

(B) the trophic parameters (as d^−1^), assigned to each FN excluding detritus (closure term for the model), which were:

- Production rate per unit of biomass (μ) (full description and references in SI);
- Consumption rate per unit of biomass (α) (full description and references in SI);
- Not-assimilated fraction of consumed biomass (ε) (full description and references in SI);
- Phototrophy/Heterotrophy ratio in individual metabolism (Ph/Het, adimensional) (full description and references in SI).

(C) trophic links among FNs and their potential weights (expressed as the fraction of daily-ration-biomass taken by a predator from a prey) (full description and references in SI).

Ranges for production (μ_i_) and consumption (α_i_) rates (d^−1^) were reconstructed from literature data, as well as the non-assimilated fraction (ε_i_) and proportions of flows to non-living nodes (γ_i_) to describe the fate of feces, deaths and excretions. All input data, except the Carbon-biomass of FNs and Ph/Het, were assumed within a range, whose amplitude depended from the oceanographic/trophic state (either blue or green) and environmental/spatial localization of the FN (either in surface or subsurface layers), as predicted by recalculation of data from the literature used as a reference (see above).

Trophic preferences for each node (δ_ij_) were at first defined on the basis of i) available literature data; ii) known ecological and biological aspects of each species or groups of species. Using the software package Ecopath with Ecosim (EwE^34^) that was specifically developed for building and analyzing food web models, we defined for each node of the food web opportune mass balance equation as in the following.

For each primary producer (p):


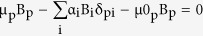


For each consumer (i):


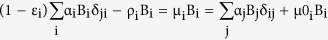


For each non-living (d):


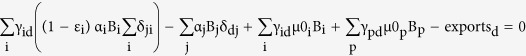


And imposing:


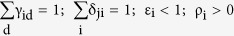


where respiration flows (ρ_i_) and natural mortality flows (μ0_i_) were estimated by the model. Based on the equations indicated above, Ecopath permits to build, under the assumption of mass balance, networks that are strongly data-driven. Although lacking of realistic dynamics, such models are snapshots of the ecosystem network that capture at best the whole set of collected data and facilitate application of comparative system analyses^42^,^43^,^56^.

The development of the detailed plankton network models followed several steps that brought to two distinguished food webs. This was achieved by MCMC application with the following rules: i) within node consistency of parameters, i.e. allowing respiration ρ_i_ > 0; ii) system overall consistency, i.e. sum of all primary productions <sum of all consumer productions; iii) trophic links were treated assuming possible variability up to 100% of initial value and links were removed when weight was <10^−4^ of the sum of links for the predator ; iv) in a final step, all trophic links, production and consumption rates were randomly changed in the +/−5% range around initial values to search for, with a MCMC procedure, the mass-balance. The above reported searches were done iteratively using MCMC balancing searching routines in EwE^75^.

## Additional Information

**How to cite this article**: D’Alelio, D. *et al*. Ecological-network models link diversity, structure and function in the plankton food-web. *Sci. Rep*. **6**, 21806; doi: 10.1038/srep21806 (2016).

## Supplementary Material

Supplementary Information

Supplementary Data 1

Supplementary Data 2

Supplementary Data 3

Supplementary Data 4

Supplementary Data 5

## Figures and Tables

**Table 1:** List of the functional nodes of the planktonic food-web, their biological properties and biomasses at the two system-states.

	Functional Nodes (FN)	Small description	Trophic status	Size (μm)	Individual Carbon (ngC)	Blue	Green
						Biomass (mgC m^-2^)	Biomass (mgC m^-2^)
1	Cyanobacteria (s)	Mainly *Synechococcus*	A	1*	3.0 10^−4^	3.2	4
2	Prochlorophytes (s)	Mainly *Prochlorococcus*	A	1*	1.0 10^−4^	0.3	0.4
3	Phyto-nanoflagellates (s)	Several species	A	1.9*	5.1 10^−3^	22	80.5
4	*Chaetoceros* spp. (s)	Diatom genus	A	2.4*	7.7 10^−3^	4.2	83.3
5	*Leptocylindrus* spp. (s)	Diatom genus	A	5.8*	6.5 10^−2^	31.3	317
6	*Skeletonema* spp. (s)	Diatom genus	A	3.1*	1.5 10^−2^	5.7	47
7	Small diatoms (s)	Several species	A	3.2*	1.5 10^−2^	4.3	34.1
8	Pennate diatoms (s)	Pennate diatoms	A	3.3*	1.6 10^−2^	1.2	11.6
9	Pseudo-nitzschia spp. (s)	Diatom genus	A	3*	1.3 10^−2^	2.3	19.9
10	Centric diatoms (s)	Centric diatoms	A	12*	0.3	19.7	83.9
11	Coccolithophores (s)	Mainly *Emiliania huxleyi*	A	4.3*	4.1 10^−2^	3.9	12.3
12	Phyto-microflagellates (s)	Several species	A	4*	4.1 10^−2^	3.9	12.9
13	Mixotrophic nanoflagellates (s)	Mainly *Ollicola vangorii*	M	1.5*	2.6 10^−3^	0.1	0.2
14	Small dinoflagellates (s)	Several species	M	4.5*	7.5 10^−2^	6.6	23.5
15	Medium dinoflagellates (s)	Several species	M	9*	0.4	4.1	13.5
16	*Myrionecta rubra* (a)	Ciliate species	M	10*	0.5	0.6	2
17	*Tontonia* spp. (s)	Oligotrichous ciliate genus	M	40*	27.0	9.5	35
18	*Laboea* spp. (s)	Oligotrichous ciliate genus	M	22*	5.0	1.8	6.5
19	*Strombidium* spp. (s)	Oligotrichous ciliate genus	M	38*	23.5	11.6	34.6
20	HNF (s)	Agglutinated nanoflagellates	H	2.4*	9.8 10^−3^	0.4	1.3
21	Hetero- dinoflagellates (s)	Several species	H	11.1*	0.8	7.7	48
22	Prostomatids (s)	Agglutinated ciliates	H	26.8*	8.8	1.7	17.5
23	*Strobilidium* spp. (s)	Ciliate genus	H	26.8*	8.8	4.3	12.9
24	Tintinnids (s)	Agglutinated ciliates	H	11*	0.7	0.2	1.7
25	Nanociliates (s)	Agglutinated ciliates	H	8*	0.3	0.7	2.3
26	Cyanobacteria (d)	Mainly *Synechococcus*	A	1*	3.0 10^−4^	108.4	155.9
27	Prochlorophytes (d)	Mainly *Prochlorococcus*	A	1*	1.0 10^−4^	10.8	15.6
28	Phyto-nanoflagellates (d)	Several species	A	1.9*	5.1 10^−3^	33.6	48.3
29	Coccolithophorids (d)	Mainly *Emiliania huxleyi*	A	4.3*	4.1 10^−2^	166.2	239
30	Diatoms (d)	Several species	A	3.2*	1.5 10^−2^	10.3	14.7
31	Mixotrophic nanoflagellates (d)	Several species	M	1.5*	2.6 10^−3^	0.1	0.1
32	Small dinoflagellates (d)	Several species	M	4.5*	7.5 10^−2^	85.5	108.2
33	Medium dinoflagellates (d)	Several species	M	9*	0.4	52.9	62.3
34	HNF (d)	Agglutinated nanoflagellates	H	2.4*	9.8 10^−3^	0.1	0.1
35	Hetero- dinoflagellates (d)	Several species	H	11.1*	0.8	34.2	44.6
36	Prostomatids (d)	Agglutinated ciliates	H	26.8*	8.8	7.3	16.2
37	*Strobilidium* spp. (d)	Ciliate genus	H	26.8*	8.8	19.1	12
38	Tintinnids (d)	Agglutinated ciliates	H	11.4*	0.7	1	1.6
39	Nanociliates (d)	Agglutinated ciliates	H	8*	0.3	3	2.1
40	Heterotrophic bacteria (s)	–	H	0.5*	n.e.	32.7	108.5
41	Heterotrophic bacteria (d)	–	H	0.5*	n.e.	373.5	397.3
42	*Penilia avirostris* (a)	Cladoceran species	H	800¯	1,670	96.1	100.8
43	Cladocerans (a)	*Evadne* & *Pseudevadne* spp.	H	900¯	1,700	33.8	65.7
44	*Paracalanus parvus* (a)	Calanoid copepod species (adults)	H	850¯	1,856	25.5	26.8
45	*Acartia clausii* (a)	Calanoid copepod species (adults)	H	1,150¯	2,852	7.5	22
46	*Temora stylifera* (a)	Calanoid copepod species (adults)	H	1,000¯	10,177	39.1	37
47	*Centropages typicus* (a)	Calanoid copepod species (adults)	H	1,000¯	6,507	12.2	24.6
48	*Other calanoids* (a)	Agllutinated genera (adults)	H	1,050¯	2,027	8.7	7.7
49	Juvenile calanoids (a)	Juveniles of calanoid copepod	H	450¯	928	14.6	21.2
50	Appendicularia (a)	Agglutinated species	H	3,000¯	3,000	36.1	39.8
51	Doliolids (a)	Agglutinated species	H	1,500¯	2,750	2	3.7
52	Salps (a)	Agglutinated species	H	10,000¯	50,200	16.2	30.8
53	Meroplankton (a)	Agglutinated larvae	H	250¯	1,643	3.5	4.7
54	*Oithona* spp. (a)	Cyclopoid copepod genus	H	675¯	404	1.4	1.3
55	Detritivora (a)	Cyclopoid copepod genera	H	650¯	2,192	7.4	5.2
56	Carnivora (a)	Mainly chaetognats	H	28,000¯	188,520	276.3	295.5
57	Appendicularia houses (a)	–	D	3,000¯	n.e.	113.8	489.9
58	Small F.P. (a)	Faeces of small animals	D	<200¯	n.e.	81.5	396.5
59	Salp F.P. (a)	Faecal pellets of salps	D	>200¯	n.e.	3.8	7.3
60	Carnivores F.P. (a)	Faecal pellets of carnivores	D	>200¯	n.e.	0.6	1.2
61	DOC (s)	Dissolved Organic Carbon	D	–	n.e.	16.6	102.9
62	DOC (d)	Dissolved Organic Carbon	D	–	n.e.	58.3	81.9
63	Generic particulate detritus (a)	Amorphous particulate detritus	D	<200¯	n.e.	4486.8	2629.7

(s) Living in the surface water-layer.

(d) Living in the deeper water-layer.

(a) Living all over the water column. A = autotrophic. H = heterotrophic. M = mixotrophic. D = detritus. *Equivalent Sphere Diameter (average). ^−^Length (average).
