# Supplementary material for: Ecological-network models link diversity, structure and function in the plankton food-web
Source: Sci Rep. 2016 Feb 17;6:21806. doi: 10.1038/srep21806 (PMC4756299; doi:10.1038/srep21806)
Supplement: Supplementary Information [file srep21806-s1.pdf]

Supplementary Information

Ecological-network models link diversity, structure and function in the plankton food-web

D'Alelio, Domenico, Libralato, Simone, Wyatt, Timothy & Ribera d'Alcalà, Maurizio

The present document includes:

Supplementary Figure 1

Supplementary Figure 2

Supplementary Figure 3

Supplementary Methods

Supplementary Figure 1

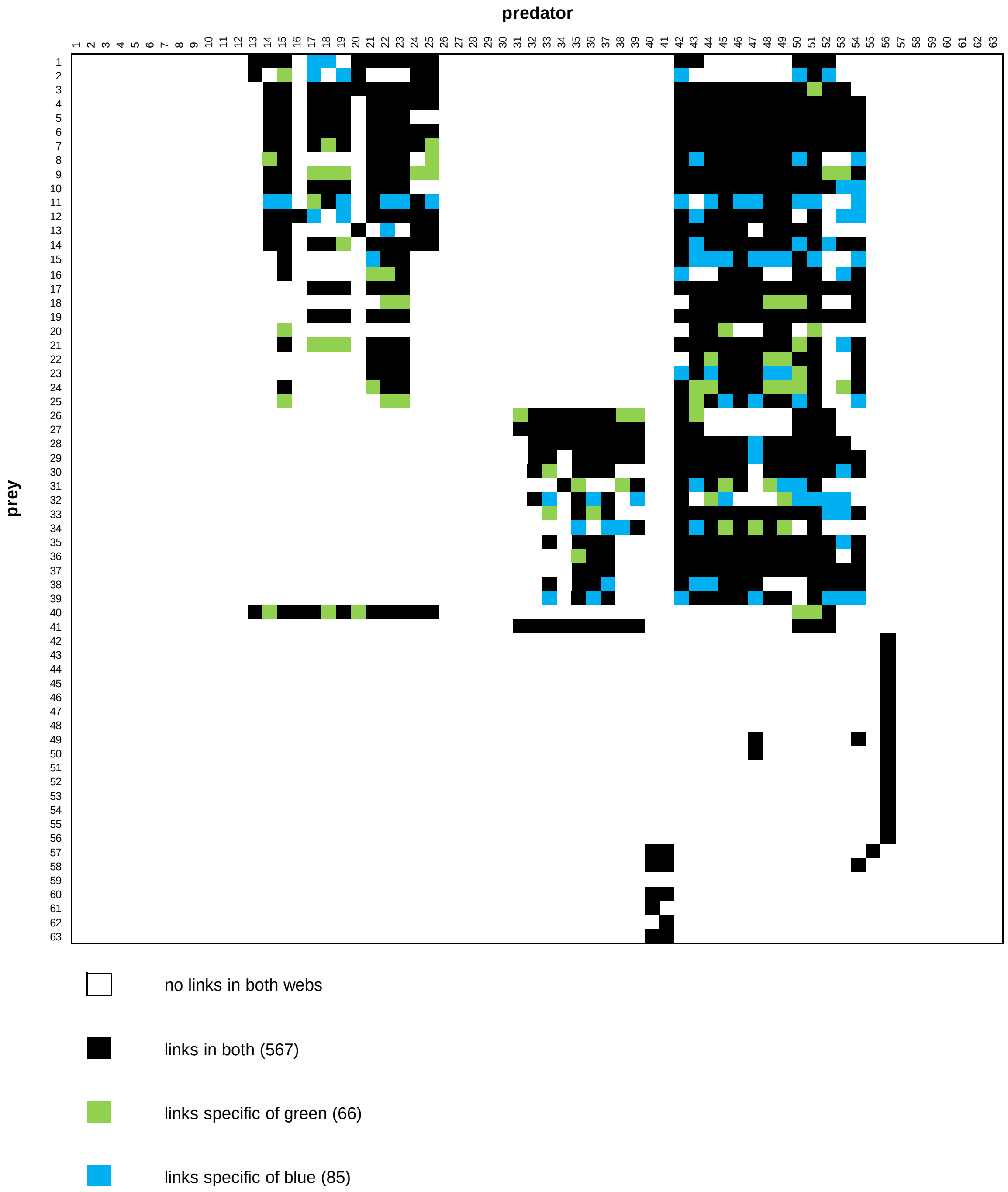

Supplementary Figure 1. Comparison between diet-matrices of Blue and Green states. Full boxes represent trophic links present after MCMC simulation. Black boxes indicate links present in both states. Blue and Green boxes indicate trophic links present in only one state.

Supplementary Figure 2

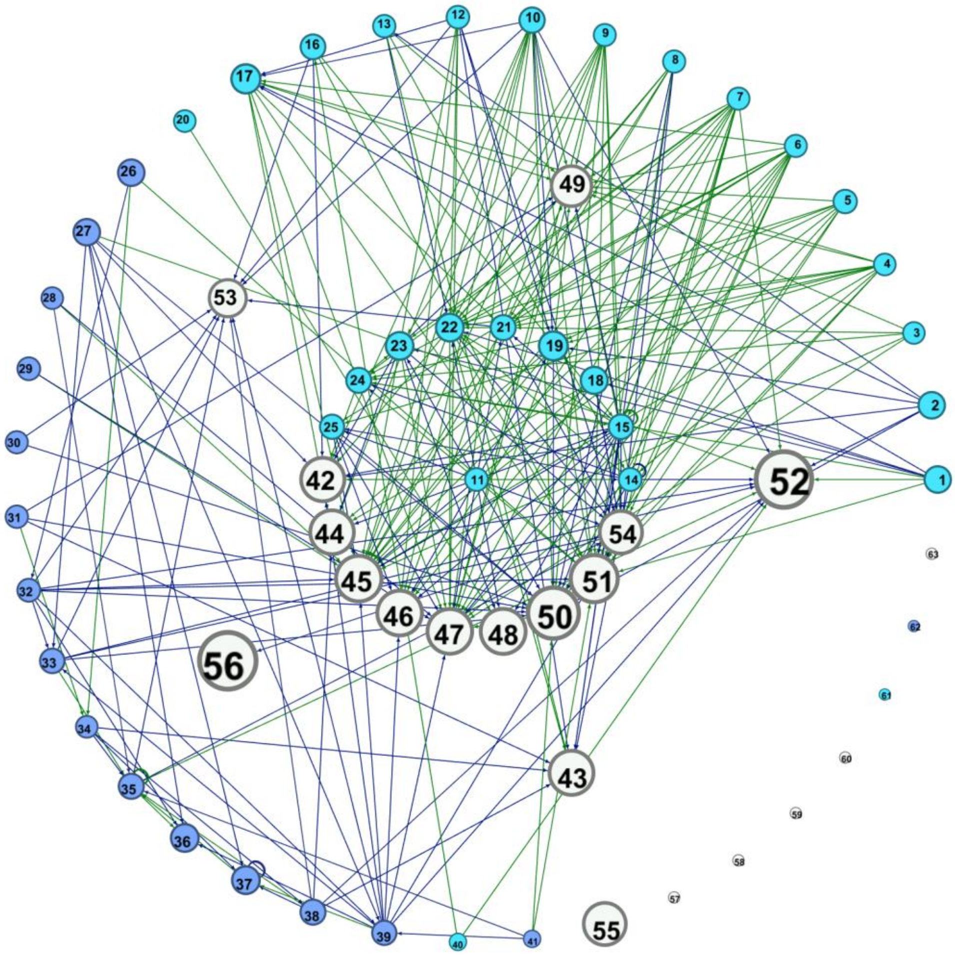

Supplementary Figure 2. Radial network combining fluxes to mesozooplankton for both green and blue food-webs. Dimensions of nodes and labels are proportional to individual size of each FN. Nodes from to 42 to 56 are mesozooplankton. All other nodes are unicellular organisms. Colors of the nodes based on water layer: cyan = FN living in the surface water layer; blue = living in the deeper water layer; white = throughout water column. Links in the network are based on the differences between matrixes of consumption in the blue and green states (B and G, respectively), calculated as (B-G)/(B+G) (Supplementary Data 4-5). Only links for which the absolute value of (B-G)/(B+G) is higher than 0.75 are shown. Blue and green links in the radial network are links for which (B-G)/(B+G) is positive (i.e. consumption higher in blue) or negative (consumption higher in green), respectively.

Supplementary Figure 3

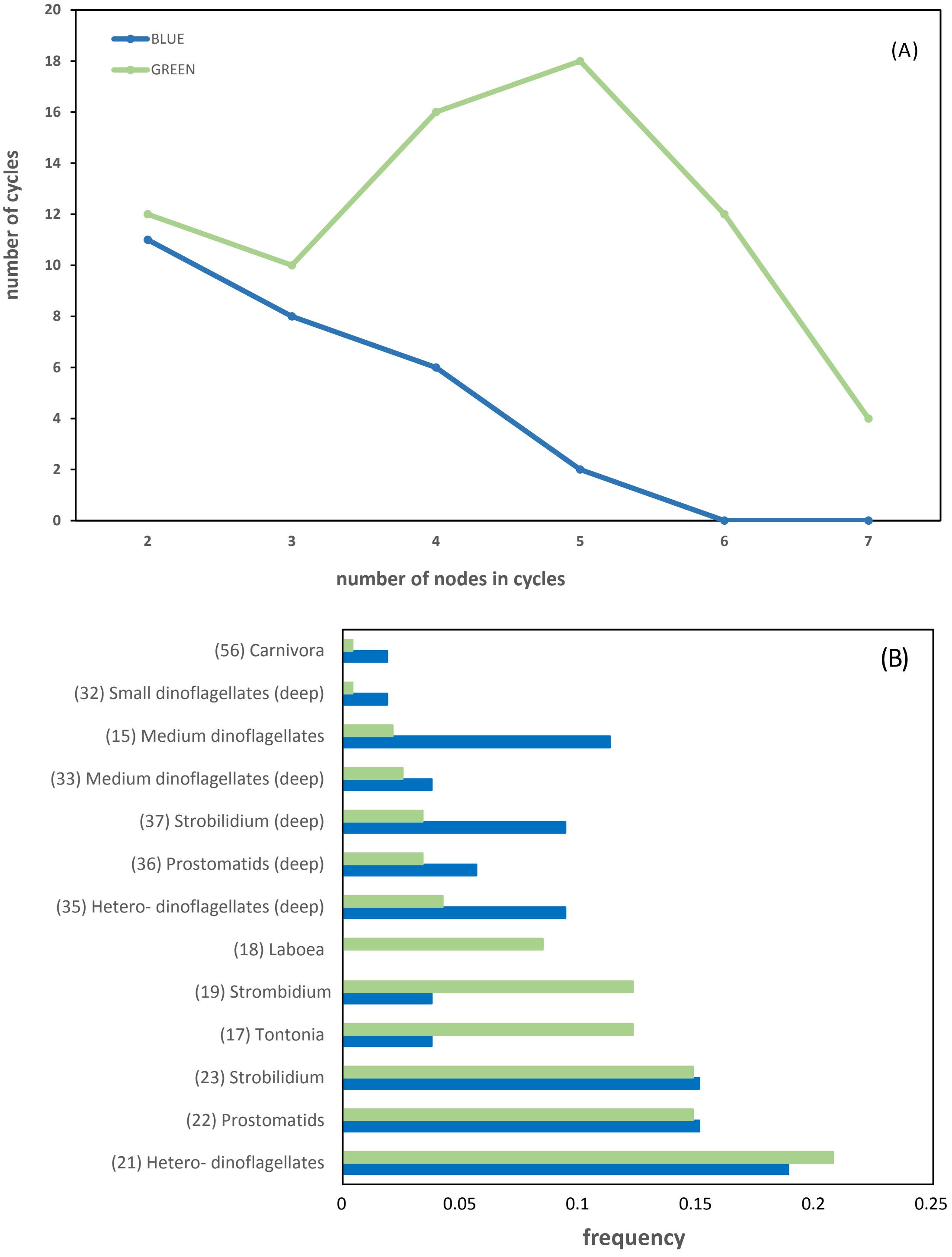

Supplementary Figure 3. Predatory cycles. Trophic pathways that start from a node and end to the same node are shown. Predatory cycles do not involve any living node (i.e. detrital forms). (A) Plot of number of cycles vs. number of nodes in cycles in the Green and Blue states. (B) Nodes taking part of cycles and quantification of their partecipation in the Green and Blue webs.

Supplementary Methods

Ecological-network models link diversity, structure and function in the plankton food-web

D'Alelio, Domenico, Libralato, Simone, Wyatt, Timothy & Ribera d'Alcalà, Maurizio

Index

1. Derivation of biomasses of Functional Nodes ... 2
1.1 Carbon biomass of phytoplankton in the surface layer ... 2
BOX 1. Definition of autotrophy/heterotrophy in the FWM ... 3
1.2 Carbon biomass of mixo- and heterotrophic protozooplankton in the surface layer ... 3
1.3 Carbon biomass of unicellular organisms in the deeper layer ... 4
1.4 Carbon biomass of heterotrophic bacteria in surface and deeper layers ... 5
1.5 Carbon biomass of mesozooplankton organisms over the water column ... 5
1.6 Carbon biomass of Appendicularia houses ... 6
1.7 Carbon biomass of faecal pellets ... 6
1.8 Carbon biomass of generic particulate detritus ... 6
1.9 Dissolved Organic Carbon ... 6

2. Derivation of production rate per unit of biomass (μ) ... 7
2.1 μ of total phytoplankton ... 7
2.2 μ of strictly phototrophic unicellular organisms in the surface layer ... 7
2.3 μ of mixotrophic unicellular organisms in the surface layer ... 8
2.4 μ of strictly heterotrophic unicellular organisms in the surface layer ... 8
2.5 μ of unicellular organisms in the deep layer ... 9
2.6 μ of mesozooplankton ... 9

3. Derivation of consumption rate per unit of biomass (α) and diet ... 10
3.1 α and diet of nanoflagellates ... 11
3.2 α and diet of dinoflagellates ... 11
3.3 α and diet of mixotrophic ciliates ... 12
3.4 α and diet of heterotrophic ciliates ... 12
3.5 α and diet of cladocerans ... 13
3.6 α and diet of calanoid copepods ... 14
3.7 α and diet of* Oithona *spp. ... 15
3.8 α and diet of Appendicularia ... 15
3.9 α and diet of doliolids and salps ... 16
3.10 α and diets of meroplankton, detritivores and carnivores ... 16

4. Derivation of Not-Assimilated fraction of Consumed Biomass (ε) ... 17

5. Supplementary References ... 18

1. Derivation of biomasses of Functional Nodes

The following simplified schematization of the overall amount of total carbon (integrated value, between 0 and -60 m) in our system at Green and Blue states was taken into account:

(Equation 1)

Total Carbon (mg m^{-2}) = C-mesozooplankton + Particulate Organic Carbon (POC) + Dissolved Organic Carbon (DOC)

(Eq. 2)

POC (mg m^{-2}, in surface or deeper layer) = C-phytoplankton + C-mixotrophic protozooplankton + C-heterotrophic protozooplankton + C-bacteria + C-detritus

POC concentration (expressed as mg m^{-3}, particle size > 0.7 µm and excluding planktonic animals) was estimated at LTER-MC at seven depths (0.5, 2, 5, 10, 20, 40, 60 m) on a weekly basis in the years 2007-2009. We took as reference for the global POC-budget data referring to years 2007 and 2009, during which the Summer-alternation between Blue and Green states was particularly evident [1].

The repartition of Total Carbon for the Green and Blue states in the surface and deeper layers in the above-mentioned main components is shown in Table 1.

1.1 Carbon biomass of phytoplankton in the surface layer

C-phytoplankton (mg m^{-2}) in Eq. 1 refers to the fraction of POC attributed to phototrophic organisms (both pro- and eukaryotic taxa) and it was derived from Chlorophyll *a* (Chl *a*) values (mg m^{-3}) by means of linear regression (Model II) obtained by plotting POC concentration (mg C m^{-3}) against the relating Chl *a* (mg C m^{-3}) values, according to:

(Eq. 3)

$$POC (z) = A \bullet Chl\ a\ (z) + B$$

In which A • Chl *a* (z) is the portion of POC at depth z associated to phytoplankton and, partially (details in the following sections), to mixotrophic protists. A is the conversion factor, while B is the portion of non-phytoplankton POC, such as bacteria, heterotrophic protists and detritus. C-phytoplankton (z) was thus derived by multiplying Chl *a* by A.

In order to obtain the conversion factor A, distinct regressions were carried out for the surface and deeper water-layers, with no distinction between Green and Blue states data. The latter option was taken based on two considerations: i) in distinct regressions on either Green or Blue data points, POC and Chl *a* were positively related, with similar trends, suggesting that Chl *a* was the main driver of POC increase at both oceanographic states; ii) due to the paucity of data, all available data were aggregated in a single plot to increase the statistical significance of the derived linear regression. The positive correlation for the surface dataset was significant ($r=0.79$; $p=10^{-13}$), and parameters for Equation 3 were: A = 94.25 and B = 122.55. A statistically significant positive correlation between POC and Chl *a* was detected for the deeper layer too ($r=0.58$; $p=10^{-8}$),for which A = 25.25 and B = 63.48.

The overall integrated amount of C-phytoplankton (expressed as mgC m^{-2}) within the surface layer was then distributed into the phototrophic FNs (FN 1-12) using the following formula:

(Eq. 4)

$$B_i\ (mg\ m^{-2}) = \text{C-phytoplankton}\ (\text{in surface layer}) \bullet RB_i \bullet [\textstyle\sum_{i=1,12} RB_i + (0.50 \bullet RB_{13} + 0.59 \bullet RB_{14} + 0.59 \bullet RB_{15} + 0.90 \bullet RB_{16} + 0.83 \bullet RB_{17} + 0.64 \bullet RB_{18} + 0.81 \bullet RB_{19})]^{-1}$$

Where B_i is the integrated biomass (0-5 m) of a generic FN *i*, RB_i is the Relative Biomass (as mgC m^{-3}) of the generic FN as derived by multiplying the average concentration of FN present at LTER-MC at 0.5 m during the studied period at either Green or Blue states based on [1] by the average cellular biomass of the taxa included in the FN (according to [2–4]). The same groups of phytoplankton as in [1] were used in our model with the exception of Cyanobacteria and Prochlorophytes (FNs 1-2), which were introduced anew. In case of

multi-specific groups, the average carbon per cell was estimated taking into account the quantitative proportion of each species in the group, based on not-aggregated abundance data at 0.5 m (not shown). The RB of prokaryotic photoautotrophs was estimated based on published works referring to GoN [5,6]. Overall, the estimation of RB_i was independent from that of C-phytoplankton, while B_i was a sort of integration between the two independent estimates. The multiplying factors in Equation 4 refer to the contribution of photo-autotrophy in mixotrophic FNs (see below). In the surface water layer, seven mixotrophic FNs were introduced, namely Mixotrophic nanoflagellates, Small dinoflagellates, Medium dinoflagellates, *Myrionecta rubra*, *Tontonia* spp. *Laboea* spp. and *Strombidium* spp., (i.e. FN 13-19). Being these organisms both autotroph and heterotroph, we considered one fraction of their biomass as 'phytoplankton' carbon (thus related to Chl *a* levels in the system) and a second fraction as included in the 'protozooplankton' carbon pool. The multiplying factors in Equation 4 represent the contribution of autotrophy to the metabolism of each *FN* (see Box 1).

BOX 1. Definition of autotrophy/heterotrophy in the FWM
(FN 13) Since no information was available in the literature regarding growth and metabolism in mixotrophic nanoflagellates (FN 13, *Ollicola vangorii*), we assumed that 50% of the biomass production in this organism was carried out via photo-autotrophy and we thus set a multiplying factor of 0.5 in Equation 4. We believe that the latter was not a strong assumption, being the contribution of mixotrophic nanoflagellates in terms of biomass extremely exiguous in comparison with other mixotrophs.
(FN 14-15) Small and medium mixotrophic dinoflagellates bear proper plastids, which are transmitted to their progeny after cell-division. Autotrophy could account for 59% of their growth rate and, consequently, of their metabolism (recalculations from data in Fig. 4 and Tables 3-4 in [7]).
(FN 16) *Myrionecta rubra* (= *Mesodinium rubrum*) is the most studied mixotroph in the plankton (e.g. [8–10]). This ciliate performs raptorial feeding at expenses of small cryptophytic microalgae (a group of phototrophs of size < 10 µm, grouped in the FN 12) and, upon ingestion of the prey, it can retain plastids for photosynthesis (i.e. kleptoplastidity, [11]). The potential contribution of ingested carbon to production in this ciliate was around the 10% [9,12].
(FN 17-19) The oligotrichous ciliates *Laboea* spp., *Tontonia* spp. and *Strombidium* spp. engulf microalgae and retain their plastids but are not able to maintain plastidial-division [12]. Aplastidic cells were rarely reported in these genera, which relied on photosynthesis to cover a large part of the respiration demand and showed growth limitation in absence of plastids [12]. On average, the overall contribution of photosynthesis to production/biomass was around 55% [12]. A meta-analysis based on published data [13] estimated that, close to light-saturation (200 microE, comparable with the light amount in the surface layer of the GoN during summer), the carbon-fixation rate in plastidic oligotrichous ciliates had an average value of 5 fg C per μm^{-3} of ciliate-biovolume *per* hour, and this rate was independent from the size of the ciliate. Based on these assessments, the fraction of biomass obtained via photosynthesis at saturating light was 0.81, 0.64 and 0.83 in *Strombidium*, *Laboea* and *Tontonia* (*FN*s 17-19), respectively, considering a summer day-length of 14 hours.

1.2 Carbon biomass of mixo- and heterotrophic protozooplankton in the surface layer

The overall amount of Carbon in protozooplankton integrated within the surface layer was estimated indirectly using the formula:

(Eq. 5)

$$\text{C-protozooplankton}_{\text{(in surface layer, mg m}^{-2}\text{)}} = \text{C-phytoplankton}_{\text{(in surface layer)}} \bullet \text{C-protozooplankton}_{\text{(at 0 m)}} \bullet \text{C-phytoplankton}^{-1}_{\text{(at 0 m)}}$$

where C-protozooplankton (at 0 m) and C-phytoplankton (at 0 m) are the sum of RBs within protozooplankton and phytoplankton, respectively, and are expressed as mg m^{-3}. The derivation in Eq. 5 stems from the assumption that protozooplankton at 0 m and the overall amount of this carbon pool in the whole layer (0-5 m) was in the same ratio than phytoplankton at 0 m and the total phytoplankton in the whole surface layer (0-5 m) (based on observations in [14]). RB_i values were obtained by multiplying the carbon per cell in each FN based on ([4]) and the abundance of the FN estimated by microscope counting (data in [1]). Being some FNs made of multi-specific groups, the average carbon per cell in each FN was estimated taking into account the quantitative proportion of each species in the group based on non-aggregated abundance data at 0 m (not shown).

Carbon allocated in a generic heterotrophic protozooplanktonic FN *i* within the surface layer (FN 20-25) was estimated as:

(Eq. 6)

B_i (mg m^{-2}) =
C-protozooplankton (in surface layer) • RB_i • $[(\sum_{k=20,25} RB_k) + (0.5 \bullet RB_{13} + 0.41 \bullet RB_{14} + 0.41 \bullet RB_{15} + 0.10 \bullet RB_{16} + 0.17 \bullet RB_{17} + 0.36 \bullet RB_{18} + 0.19 \bullet RB_{19})]^{-1}$

where the multiplying factors are the complement to 1 of multiplying factors in Equation 4 and RB_i were derived as for Equation 4.

Carbon in a generic mixotrophic protozooplanktonic FN *i* within the surface layer (FN 13-19) was given by the following equation:

(Eq. 7)
B_i (mg m^{-2}) =
{C-phytoplankton (in surface layer) • $(\boldsymbol{a} \bullet RB_i) \bullet [(\sum_{k=1,12} RB_k) + (0.50 \bullet RB_{13} + 0.59 \bullet RB_{14} + 0.59 \bullet RB_{15} + 0.90 \bullet RB_{16} + 0.83 \bullet RB_{17} + 0.64 \bullet RB_{18} + 0.81 \bullet RB_{19})]^{-1}$}
+
{C-protozooplankton (in surface layer) • $(\boldsymbol{b} \bullet RB_k) \bullet [(\sum_{k=19,23} RB_k) + (0.50 \bullet RB_{13} + 0.41 \bullet RB_{14} + 0.41 \bullet RB_{15} + 0.10 \bullet RB_{16} + 0.17 \bullet RB_{17} + 0.36 \bullet RB_{18} + 0.19 \bullet RB_{19})]^{-1}$}

where $\boldsymbol{a}$ = 0.5, 0.59, 0.59, 0.90, 0.83, 0.64, 0.81 and $\boldsymbol{b}$ = 0.50, 0.41, 0.41, 0.10, 0.17, 0.36, 0.19 when *i* went from 13 to 19, respectively. These multiplying factors were derived according with information in Box1. RB_i values were derived as for Equation 4.

1.3 Carbon biomass of unicellular organisms in the deeper layer

The total amount of phytoplankton in the deeper layer (C-phytoplankton (in deeper layer)) was obtained by converting direct measurement of Chl *a* across that water-layer. Yet, protozooplankton biomass in the deeper layer was inferred following the same criterion used for the surface layer:

(Eq.8)
C-protozooplankton (in deeper layer, mg m^{-2}) =
C-phytoplankton (in deeper layer) • C-protozooplankton (0 m) • C-phytoplankton^{-1} (at 0 m)

where C-protozooplankton and C-phytoplankton are expressed as mgC m^{-3}. We assumed that, in the deeper layer, the ratio between phyto- and protozooplankton was the same as in the surface layer.

In the deeper layer, we assumed that the concentrations of unicellulars detected at one single depth were representative of the whole water layer, stemming from the observation that the water below the seasonal thermocline was permanently mixed in the GoN during summer [14]. The repartition of biomass among unicellulars in the deeper layer was carried out using the following formulas:

(Eq. 9)
B_i =
C-phytoplankton (in deeper layer) • $RB_i \bullet [(\sum_{k=26,30} RB_k) + (0.50 \bullet RB_{31} + 0.59 \bullet RB_{32} + 0.59 \bullet RB_{33})]^{-1}$
(With i going from 26 to 30 (phototrophic FNs))

(Eq. 10)
B_i =
{C-phytoplankton (in deeper layer) • $(\boldsymbol{a} \bullet RB_i) \bullet [(\sum_{k=26,30} RB_k) + (0.50 \bullet RB_{31} + 0.59 \bullet RB_{32} + 0.59 \bullet RB_{33})]^{-1}$}
+
{C-protozooplankton (in deeper layer) • $(\boldsymbol{b} \bullet RB_i) \bullet [(\sum_{k=34,39} RB_k) + (0.50 \bullet RB_{31} + 0.41 \bullet RB_{32} + 0.41 \bullet RB_{33})]^{-1}$}
(With i going from 31 to 33 (mixotrophic FNs))

(Eq. 11)
B_i =
C-protozooplankton (in deeper layer) • $RB_i \bullet [(\sum_{k=34,39} RB_k) + (0.50 \bullet RB_{31} + 0.41 \bullet RB_{32} + 0.41 \bullet RB_{33})]^{-1}$

(With i going from 34 to 39 (heterotrophic FNs))

In Equation 10, ***a*** = 0.5, 0.59, 0.59 and ***b*** = 0.50, 0.41, 0.41, for FNs from 31 to 33, respectively (as in former sections).

Referring to Equations 9-11, the relative biomass (RB) of the two prokaryotic FNs in the deeper layer (FN 26-27) was calculated by multiplying the abundances (derived from [5]) by the carbon cell quota (the same as for FNs 1-2). The RB of nanoplankton (including only eukaryotic cells) at station LTER-MC during summer were derived from [15]. Within the total nanoplankton concentration, 30% was represented by coccolithophores (FN 29; same carbon-per-cell quota as for FN 11), while diatoms were the least abundant group, accounting for about 5% of the total (FN 30; carbon-per-cell quota as the median value of FNs 4-10).

Only three categories of mixotrophs were introduced in the deeper layer (FNs 31-33; same carbon-per-cell quota as for FN 13-15), with the exclusion of plastidic ciliates. It is plausible that the latter occur mainly in the surface mixed-layer of marine systems, being them i) limited by plastid-providing food and light and ii) not able to slow down their respiration rates in absence of light ([12] and references therein).

The definition of biomass of *Myrionecta rubra* (FN 16) needs a specific discussion. This species was also excluded from the deeper layer biomass budget, based on the fact that *M. rubra* is able to migrate daily for several tens of meters along the water column and to set in the surface mixed-layer at mid-day ([12] and references therein). Since sampling at LTER-MC was usually carried out at that time of the day, we hypothesized that sampled *M. rubra* populations at our long-term station were at the same concentrations at which they could be found at depth during other periods of the day. Thus, contrarily to other protists, which were divided into two distinct populations (namely, surface and deep), despite the time of the day, we considered *M. rubra* as the only unicellular organism with a biomass distributed along the whole water-column, alike for mesozooplankters (see next sections).

Abundances of mixo- and heterotrophic dinoflagellates in the deeper layer (FN 32-33 and FN 35, same cell carbon as for FN 13-14 and FN 21) were derived from [15], who reported that dinoflagellates accounted for about 15% of the total abundance at 25 m in the GoN during summer. We sub-divided this amount among the three categories of dinoflagellates already considered in the surface layer (two of them being mixotrophic, one being heterotrophic), but proportionally to the abundance ratios among these three categories in the surface layer. RB for these FNs was derived as for the other FNs, thus multiplying abundance by carbon-cell quota.

The abundances of other heterotrophic protists in the deeper layer (FN*s* 36-39, same cell carbon as in FN 22-25) were derived using the abundance of heterotrophic dinoflagellates (FN 35) as a reference. Heterotrophic dinoflagellates in the deeper layer below the seasonal thermocline accounted for <15% of the total of dinoflagellates at 25 m in the GoN during summer [15]. Since data were not available for the other heterotrophic protozooplankters, their abundances were mathematically derived assuming that the abundances of heterotrophic protozooplankon in the deeper layer were in the same proportions as the abundances of these organisms in the surface layer. Once estimated the concentrations, RB for these FNs was derived as for the other FNs.

1.4 Carbon biomass of heterotrophic bacteria in surface and deeper layers

Heterotrophic bacteria were divided into surface and deeper layers' populations (FN 40-41). These quantities were derived by regression from Chl *a* data, according to [6], since a strict positive co-variation was described between these two POC compartments in the GoN. Bacterial cells concentrations were converted in units of biomass (mg m^{-3}) by multiplying them by 0.020 pg C, i.e. the carbon-per-cell-quota for marine bacteria [16]. The discrete values were then integrated through the surface and the deeper layers taken separately, to get absolute units of biomass (mgC m^{-2}).

1.5 Carbon biomass of mesozooplankton organisms over the water column

Mesozooplankton in our model was considered free to move along the water column throughout the thermocline. Biomasses of mesozooplankton FNs (42-56) were estimated by multiplying the average abundance of each FN at either Blue or Green states [1] by i) the carbon-per-individual quota for the same FN

as derived from published data [17–35] and ii) the vertical extension of the water-layer within which these organisms were sampled at LTER-MC (50 m) [14]. In case of multi-species FNs, the average carbon per individual was estimated taking into account the quantitative proportion of each species based on not-aggregated abundance data (not shown). Differently from [1], pelagic tunicates were divided into Doliolids and Salps (FN 51-52) due to large differences in their dimensions and biomasses [36].

1.6 Carbon biomass of Appendicularia houses

In the course of their life, Appendicularia build organic (mucous) 'houses' external to the animal body [36]. Once got engulfed by filtering activity, house are discarded by the animal that eventually secrete a new house and start feeding again. Appendicularia houses are thus a form of detritus characterized by a high nutritional value, rich of undigested microbial food, and they are eaten by a particular form of copepods (FN 55). The house renewal rates is independent from food concentration and ranges between 4.90 and 11 times the animal biomass per day, at food-concentrations spanning 60-2400 µg C L^{-1} (data recalculated from [26]), with the minimum values being in the range of those found in our system. To avoid overestimations, we kept the most conservative values and estimated the biomass of houses in our system by multiplying Appendicularia biomass by 4.90 for both Blue and Green states.

1.7 Carbon biomass of faecal pellets

The amount of faecal pellets, as units of carbon, is proportional to the assimilation efficiency of an animal – i.e. the fraction of the carbon ingested which is utilized for respiration and growth. The complement to 1 of the latter carbon represents the carbon 'egested' as faeces. We distinguished between eight different classes of faecal pellets, namely those from calanoid copepods, juveniles of calanoid copepods, cladocerans, doliolids, salps, *Oithona* spp. (no distinction between early and mature life-stages), Detritivora and Carnivora. These groups were further agglutinated into three groups, large and slow-sinking faecal pellets (Carnivora [37]), large and fast-sinking faecal pellets (Salps [36]) and small faecal pellet (including all the others) (FN 60, 59 and 58, respectively). Differently from other biomass quantities in our model, faecal pellets where linked to rates of ingestion and assimilation of animals (see next sections).

The amount of faecal pellets present in the system (expressed as as mg m^{-2}) was calculated as:

(Eq. 12)

$$B_{\text{faecal pellet}} = B_{\text{animal}} \bullet (\alpha)_{\text{animal}} \bullet (\varepsilon)_{\text{animal}}$$

where α and ε were the consumption rate and the fraction of consumed biomass not assimilated by the animal, respectively (see next sections). Since the assimilation efficiencies of animals depends to some extent from the food available, the values for these ratios were not equivalent in the Green and Blue food-webs and thus also the amount of faecal pellets present in the system was different between these states. Moreover, since α and ε were represented by a range of variability (and not by a single value, see next sections dealing with production and feeding rates) in each of the system states, in calculating faecal pellets amounts, we used a single value for these rates, intermediate within the range of variability indicated above. Finally, assuming a fast physical degradation of large but loose faeces of chaetognats (Carnivora), the calculated amount of Carnivora faecal pellets were reduced to the 10% percent and the remnant 90% was assigned to generic particulate detritus.

1.8 Carbon biomass of generic particulate detritus

This quantity (FN 61) was derived by subtracting biomasses of all faecal pellets and Appendicularia houses from C-detritus (derived from Eq. 2); the latter included all uneaten and dead organic carbon derived from FNs mortality. The flow-to-detritus matrices for Green and Blue states are shown in Dataset 3.

1.9 Dissolved Organic Carbon

This component was assumed to be the 13% of the phytoplankton biomass, according to recalculations from [38] (see also details in the next section). DOC was divided between surface and deep layers (FNs 62-63).

2. Derivation of Production rate per unit of biomass (μ)

Values of Max-Min μ before MCMC and the final post-MCMC value are presented in Dataset 1.

2.1 Total phytoplankton μ

Primary production data collected at the LTER-MC almost weekly during summer months of 1984-1988 were analysed. The sampling was carried out as illustrated in [14], at 0.5, 2, 5, 10, 20, 30, 40, 50, 60 m of depth. The particulate primary production (PP) was estimated using a standard ^{14}C method. The integrated particulate primary production (PP) for the two layers (0-5 and 5-60 m in depth) was obtained by computing discrete values. The ^{14}C method estimated only PP but we inferred also the amount of exudates release (ER) to get the total primary production (TP), which is the sum of PP and ER. According to [38], ER accounted for 13% of total fixed carbon, on average (i.e. including all aquatic systems - recalculated data). By using the linear regression included in [38] with parameters derived for marine systems, the total primary production in our system was gained by the formula:

(Eq. 13)

$$TP_{\text{(in surface or deeper layers)}} = PP_{\text{(in surface or deeper layers)}} + 10\text{^}(-0.88 + 1.03 \cdot \log_{10}(PP_{\text{(in surface or deeper layers)}}))$$

μ for surface and deeper layers were obtained by dividing TP by the total phytoplankton biomass (TB) estimated from Chl *a* with methods described in section 1.1. In order to estimate μ for Green and Blue states separately, cluster analysis on salinity and Chl *a* values for the dates considered for TP estimation was carried out, following the rationale presented in [1]. Maximum, minimum and Median values for each phase and within each water-layer were calculated. In the Green state, in the surface and deeper layers, Max, Min and Median accounted for, respectively, 0.99, 0.39, 0.63 and 0.96, 0.14, 0.29 d^{-1}. In the Blue state, in the surface and deeper layers, Max, Min and Median accounted for, respectively, 0.85, 0.22, 0.52 and 0.87, 0.17, 0.48 d^{-1}. μ rate for phytoplankton FNs was set using as reference-values both data from the literature pertaining each FN and μ estimated for the whole community based on ^{14}C and shown above. Moreover, we assumed that μ for photosynthetic FNs was equivalent to individual growth rates. Since the latter showed considerable intra-specific variability and were not completely coupled to available resources, we set the same range of variability of μ for the Green and Blue food-webs, considering that only small differences in productivity were detected between these two states.

2.2 μ of strictly phototrophic unicellular organisms in the surface layer

The maximum in-situ growth rates for *Synechococcus* (FN 1) was 1.2 d^{-1} during August [39]. Concerning Prochlorophytes (FN 2), maximum in-situ and in-vitro growth rates accounted for 0.6931 d^{-1} and 0.85 d^{-1} ([40] and Chisholm unpublished data).

Growth rates for eukaryotic phytoplankters were taken from a recent review [41]. Phyto-nanoflagellates (FN 3) included mainly Chlorophyceae having a maximum growth rate of 1.9 d^{-1}. Many surface-FNs in our model were composed of diatoms (FN 4-10), having a maximum growth rate of 2.25 d^{-1} [41]. Since maximum growth rate was inversely related to diatom biovolume [41], maximum growth rates for diatom-FNs were obtained following the allometric relation:

(Eq. 14)

$$\text{Log}_{10}(\mu\ (\text{FN diatom})) = -0.25 * \text{Log}_{10}(\text{biovolume (FN diatom)}) + 0.75$$

derived from data in figure 3D in [41].

The maximum growth rates of surface Coccolithophores (FN 11) was 1.7 d^{-1} [41]. Concerning Phyto-microflagellates (FN 12), a heterogeneous class including chlorophytes and cryptophytes, we chose as maximum growth rate an intermediate value (1.5 d^{-1}) between those of these two groups according to [41].

The minimum μ for a generic photosynthetic FN *i* in the surface layer (1-12) was chosen taking into account the minimum value of primary production detected in the surface layer and during the specific state of the system (i.e. either Green or Blue), using the following equation:

(Eq. 15)
$$\text{Min } \mu_i = [\text{Max } \mu_i] \bullet [\text{Min } \mu(\text{all phytoplankton})] \bullet [\text{Max } \mu(\text{all phytoplankton})]^{-1}$$

In which Min and Max μ for all phytoplankton were values reported in section 2.1.

2.3 μ of mixotrophic unicellular organisms in the surface layer

Differently from 'phototrophic' protists, ciliates are hard to cultivate and their physiology is largely unknown. The Max μ for mixotrophic nanoflagellates (FN 13) was set as for Phyto-nanoflagellates (FN 3), due to scarce background knowledge. The Max μ for mixotrophic dinoflagellates (FN 14-15) was set using the regression relating growth rate to metabolic state and cell-size reported by [7], resulting in values of 1.17 and 1.02 d^{-1} for smaller and larger mixotrophic dinoflagellates (FN 14-15), respectively.

Myrionecta rubra (FN 16) can build considerable biomass in coastal-transition systems mainly at low salinity (about 10 PSU, [10]). In saltier waters, the maximum growth rate was 0.52 d^{-1} in culture at 31 PSU [42]. Since it is plausible that the inverse relation between the growth rate and salinity is not necessarily linear, we set the Max μ of *M. rubra* at 0.52 d^{-1}, assuming this was the highest growth rate in marine coastal waters, even though salinity in our system of study was usually higher (around 37 PSU).

Scanty information was available for mixotrophic oligotrichous ciliates in the genera *Tontonia*, *Laboea* and *Strombidium* (FN 17-19). The maximum growth rates for these were calculated according to the regression deriving maximum growth for mixo- and heterotrophic ciliates as depending from temperature and cell volume [43], considering a temperature of 25°C (average value for the surface layer in the GoN during summer). The Max μ were 1.33, 1.54 and 1.35 d^{-1} for *Tontonia*, *Laboea* and *Strombidium* (FN 17-19), respectively. These data were in line with those reported in specific studies (e.g., [44]).

The Min μ values for these mixotrophic protists was calculated by applying Equation 15, assuming a positive relation between mixotrophic productivity and that of all phytoplankton.

2.4 μ of strictly heterotrophic unicellular organisms in the surface layer

We set five FNs as prevalently heterotrophic, namely HNF (Heterotrophic Nano-Flagellates), Heterotrophic dinoflagellates, Prostomatids, *Strobilidium* spp., Tintinnids and Nanociliates (FN 20-25).

As for HNF (FN 20), a Max in-situ growth rate of 1.76 d^{-1} was reported in nature [45].

Using the regression for growth rate in dependence to cell-size presented in [7], the value of Max μ for FN 21 (Equivalent Sphere Diameter, ESD = 22 μm) resulted 0.95 d^{-1}.

Growth rate data for prostomatids (FN 22, cell size > 20 μm) were scarce in the literature. The physiological and feeding response under a monospecific diet were studied for the prostomatid ciliate *Tiarina fusus* and values of Max growth rate at 19°C were reported to range 0.1-0.47 d^{-1}, depending on the typology of prey [46]. By applying a Q_{10} of 2.8 – an average for ciliates, according to [47] – the Max μ at 25°C resulted 0.98 d^{-1}.

Being information in the literature scarce for this organism, the maximum growth rate for the heterotrophic oligotrich *Strobilidium* spp. (FN 23) was derived using equation indicated for heterotrophic oligotrichous ciliates by [43]. The Max μ at 25°C was 1.64 d^{-1}.

A wider literature was available for growth rates of Tintinnids (FN 24). In a two-years observation, the Max growth rate resulted 1.66 d^{-1} for the whole tintinnids' community during summer [48].

Nanociliates (FN 25, cell size < 20 micron) were a wide group in terms of taxonomy, including both prostomatids and other ciliates. Information about their growth performances were scarce and we took data from [49], which reported, for a nanociliate assemblage, under laboratory conditions and relaxed intra-guild predation, Max growth rates up to 2.36 d^{-1}.

The Min μ for all *FN* within heterotrophic protozoplankton was calculated with Equation 15, assuming that production in these organisms was in direct relation with production in photosynthetic organisms.

2.5 μ of unicellular organisms in the deep layer

The Max and Min μ for each unicellular FN, except diatoms, were derived using the following formulas, assuming that specific μ were correlated with μ of all phytoplankton in the deeper water-layer:

(Eq. 16)

Max μ(FN in deeper layer) = Max μ(corresponding FN in surface layer) • (Median μ(all phytoplankton in deeper layer)) • (Median μ(all phytoplankton in the surface layer))$^{-1}$

(Eq. 17)

Min μ(FN in deeper layer) = Min μ(corresponding FN in surface layer) • (Median μ(all phytoplankton in deeper layer)) • (Median μ(all phytoplankton in the surface layer))$^{-1}$

Concerning diatoms in the deeper layer (FN 30), the first terms in equations 16 and 17 were Median (Max μ (FN 4-10)) and Median (Min μ(FN 4-10)), respectively. Median μ for all phytoplankton community were taken from section 2.1.

2.6 μ of mesozooplankton

Life history of the cladoceran *Penilia avirostris* was extensively studied in the Mediterranean Sea and along Brazilian coasts [50–52]. This animal (FN 42 in our model) showed extremely high population growth pursued by parthenogenetic reproduction during summer [50,51]. Juveniles of cladocerans were mini-adults, occupied a niche comparable to the latter and grew very rapidly to the adult stage (<3 days). In our model, the Max-Min μ values for *P. avirostris* were set assuming that population increasing was dependent only on the animal birth rate and the main boost to population growth came from parthenogenetic eggs' hatching, instead of from somatic growth, alike in other planktonic animals (e.g. copepods). Since the relation between growth rates and food-environment was not studied in detail for this species, one and a single μ interval, derived from the above-mentioned studies, was considered for both Green and Blue states (growth rate ranging 0.10-1.37 d^{-1}). Yet, scanty information was available in the literature for Mediterranean *Evadne* spp. and *Pseudevadne tergestina*, the other cladocerans included in our model (FN 43). We thus assumed that Min and Max μ for these animals were the same of those set for *P. avirostris*, in both Green and Blue states.

Differently from cladocerans, copepod life-histories included several different stages. Population/individual growth rate was a good proxy of these animals’ productivity (e.g., [53]). Copepods’ μ was parameterized by the weight-specific fecundity, assuming that adult-female production was completely realized by means of egg production, without considering any changes in individual body-biomass in adults at short-time scales [54]. Yet, juvenile-copepods' μ was represented by the weight-specific growth, since early stages underwent changes in individual biomass at short time-scales [54]. μ for both adults and juvenile copepods in our FMW were set according to an extensive synopsis of growth rates of copepods and by using mathematical relations presented therein and linking growth rates to i) body-biomass of the species, ii) temperature and iii) food concentration [54]. Since copepods occurred all over the water column in our FWM, we set Max-Min μ values as the rates predicted for temperatures and food conditions present in the environment at surface-deeper layers in the Green and Blue states, respectively (T ranging 25-15°C and food ranging 203-21 and 37-17 mgC m^{-3}, respectively). Being Juvenile copepods in our model a mixture between the two genus *Paracalanus* and *Clausocalanus*, rates averaged from those referring to the latter were compiled.

Unlike cladocerans and copepods, pelagic tunicates, including Appendicularia, Doliolids and Salps (FN 50-52), reproduce only once in a lifetime (they are semelparous) and the organisms usually die after reproducing [36], they have no larval stages and newly born individuals have feeding performances and ecological role comparable to adults and, in some cases, a relatively large body-size ([36,55] and reference therein). Nonetheless, adults undergo an intense somatic growth, which could be anyway contemplated when setting the μ. The growth rate of pelagic tunicates has been represented by the so-called 'lifetime fitness', a parameter that in population ecology studies is commonly used as synonym of 'recruitment' [55]. Lifetime fitness expresses the maximum intrinsic rate of natural increase and it is determined as ln(egg produced per animal)/(development time from fertilization of an egg to spawning by the organism hatched from that egg). Thus, the lifetime fitness integrates both the timing of egg release and the somatic growth

that precedes spawning. This parameter was, to our opinion, thoroughly suitable to model μ in pelagic tunicates at the time scales of our model.

The growth performances of Appendicularia (FN 50) have been extensively explored at laboratory conditions. A life-cycle-based model was built for *Oikopleura dioica* - that is the main appendicularian species present in the GoN during summer [29] (plus unpublished data presented in [55]): therein, lifetime fitness was strongly influenced by both temperature and food concentration. Appendicularia, and pelagic tunicates in general, are particularly sensitive to high food concentrations: being them strong filter-feeders, they easily undergo super-saturation/engulfment, with negative effects on reproduction. Food availability to Appendicularia in our system do not exceeds 200 mg C m^{-3} and these animals remain well below the saturation threshold (> 300 mg C m^{-3}). By taking *O. dioica* as a reference [29], we set Max-Min μ of Appendicularia in our model (FN 50) at 0.5-0.0 d^{-1} in the Blue state and 1.125-0.0 d^{-1} in the Green state.

Among Doliolids (FN 51), the genus *Doliolum* was the most represented in the GoN during Summer and included mainly the species *D. nationalis*, which features a bi-phasic life-cycle in which asexual production of new individuals could strongly shorten the generation time [36]. Growth rates of doliolids was studied less extensively than Appendicularia. A lifetime fitness of 0.26 d^{-1} at 20°C was estimated for *D. gegenbauri* [55], that also showed a limited dependence from the food-concentrations [56]. Assuming a linear dependence between temperature and growth rates in the range 15-25°C, this parameter was 0.20-0.33 d^{-1} between 15 and 25°C (based on Q_{10} assumed for pelagic tunicates [57]). This rate referred to the complete life-cycle of *D. gegenbauri*, encompassing different life stages (i.e. up to six). Yet, *Doliolum nationalis* was capable to run a further single-stage cycle thanks to asexual reproduction. Since no estimation of growth rates for *D. nationalis* has been done so far, to our knowledge, we decided to assign a Max μ of ~1.9 d^{-1}, assuming that, during the short cycle, Doliolids had growth rates similar to those of Salps, also capable of asexual growth [36]. Due to the uncertainties in the definition of the growth-rate range in Doliolids (FN 51), we assigned Max-Min μ values of 1.9-0.20 d^{-1} in both Green and Blue food-webs.

Information about growth rates of Salps (FN 52) was even scantier. For *Thalia democratica*, the most represented species in the GoN during summer, the lifetime fitness derived from different published works ranged 0.18-1.9 d^{-1} in a range of temperature of 14-22°C, with the lower values measured in the lab and the highest ones estimated from the field ([55] and references therein). Nonetheless, a 1.9 d^{-1} could theoretically occur only in particular circumstances, such as massive salp outbreaks in nature ([55] and references therein). Yet, lower values of growth rate were probably biased by the confinement of animals in a limited space in the laboratory. Considering these issues, we decided to not take any assumption in establishing salps' μ and we considered the whole range of variability of their lifetime fitness, setting Max-Min μ at 0.18-1.9 d^{-1}, for both Green and Blue states.

Meroplankton (FN 53) included planktonic larvae of benthic organisms with life-cycles longer than those of holoplankters in general. Assuming that eggs and larvae of benthic organisms were always present in the summer plankton of the GoN, we used the rate of somatic growth as the unique proxy of μ for meroplankton, since we were not interested in the all lifetime fitness (i.e. recruitment) of these animals. Growth rates ranging 0.025-0.15 d^{-1} were reported for larvae of the polychaete *Polydora ciliata* at 16°C in the laboratory [58], with the highest rates gained at food saturation, i.e. at food concentration of about 1 mg C L^{-1}, a value about double the highest concentration of POC in our system of study. Applying the 2.8 Q_{10} by [47], the rates were ranging 0.07-0.42 d^{-1}. Due to the scarce knowledge on the relation between growth, food and temperature in meroplankton, we assigned an identical Min-Max μ range (0.025-0.42 d^{-1}, respectively) in both Green and Blue states.

No information was gathered for the growth rates of Carnivora mesozooplankton - i.e. chaetognats (FN 60). μ were interpolated by Ecopath based on these animals' consumption rates.

3. Derivation of Consumption rate (α) and diet

For each FN, Max-Min α ratios were selected, in each of the two states (i.e. Green and Blue), as derived from mass-specific ingestion rates taken and recalculated from the literature. In case of mixo-/heterotrophic unicellular organisms present in the model in two distinct populations, in either surface or deeper layers, Max-Min α were indicated for each layer. Diet-matrices were built considering information from the literature. Yet, since feeding preferences were important in defining biological differences among

FNs, in our model, we set both the overall spectrum of food eaten and a tentative food-selectivity based on expert knowledge. We also set a % of variability to the strength of each trophic link depending on the strength of the specific background information. As for other trophic parameters, α values and trophic link-weights for the final input data of the model were defined by means of uncertainty analyses via a MCMC procedure performed at the level of the all web and considering all organisms involved. Values of Max-Min α before MCMC and final value after MCMC are presented in Dataset 1. Diet-matrices before and after MCMC are presented in Dataset 2.

3.1 α and diet of nanoflagellates

Few information were found about specific ingestion rates of mixotrophic flagellates, mainly *Ollicola vangoorii* (FN 13 and FN 31, in surface and deeper layers, respectively), present in our FWM. We assumed that Max-Min α in FN 13 and FN 31 were at least twice the Max-Min μ, at both surface and deeper layers. Bacterivory was the dominant feeding behaviour in this nanoflagellate [59].

Few studies were available in the literature concerning feeding activities of Heterotrophic Nano-Flagellates (in our model, FNs 20 and 34, at surface and deeper layers, respectively). In our model, Max-Min α for HNF were set at 1.8-0.3 d^{-1}, based on Callieri et al. [60]. Since the latter study was conducted over a wide temperature range, i.e. 9-20°C, we assumed that the α-range above was valid for both surface and deeper layers. Concerning diet, HNF assumed 12-60% from heterotrophic bacteria, 0.6-19% from *Synechococcus* and 0.02-21% from *Prochlorococcus* [61]. Some HNF species also grazed pico-eukaryotes with a size <3 µm [62]. The latter were partially represented in our system of study within FNs 3 and 28. We included in HNF diet all these food-items, giving however much relative weight to trophic links involving prokaryotes.

3.2 α and diet of dinoflagellates

α for dinoflagellates was set based on meta-analyses of experimental data and relating regressions [7]. Smaller- and medium-sized mixotrophic dinoflagellates (in our model, showing an ESD of 9 and 18 µm, respectively, and included in FN 14-15 for the surface and FN 32-33 for the deeper layer) showed a Max α of 6.69 and 2.205 d^{-1} respectively. Obligate heterotrophic dinoflagellates (in our model, showing an ESD of about 22 µm and included in FN 21 and 35 for surface and deeper layer, respectively) showed a Max α of 7.674 d^{-1}. The calculated Min α were 1.338 and 0.22 d^{-1} for smaller and larger mixotrophic dinoflagellates and 1.279 d^{-1} for heterotrophic dinoflagellates. Since it was not possible to disentangle the effect of temperature on the ingestion rate from the information available in the literature, the α ranges described above were set for both water layers considered in our model.

Concerning dinoflagellates' diets, these organisms ate within a wide array of prey, according to meta-analysis on published data [7,63]. The prey of small mixotrophic dinoflagellates (FN 14 and 32) spammed from bacteria and prochlorophytes (ESD < 1 µm) to other small-sized dinoflagellates (ESD within 10 µm; cannibalistic feeding) and diatoms. The prey-range of larger-sized mixotrophic dinoflagellates (FN 15 and 33), in turn, included all other unicellular organisms, except large ciliates, which showed a cell-size apparently out of the range of mixotrophic dinoflagellates' feeding (ESD > 40 µm, in our model). Heterotrophic dinoflagellates (FN 21 and 35) ate over a prey-range wider than that for larger-sized mixotrophic dinoflagellates. Dinoflagellates' diet in our FWM was further improved by using information about the optimal-prey size for these organisms in laboratory conditions [7]: i.e. mixotrophic and heterotrophic dinoflagellates showed better growth performances when predator-prey-size ratios ranged 0.8-5.8 and 0.4-5.3, respectively. Moreover, a further weight to trophic links was set based on the observation that the higher ingestion rates were got when predator to prey size ranged 0.8-1.4 and 0.9-2.6 for mixotrophic and heterotrophic dinoflagellates, respectively [7]. The identical diet was assumed for both surface and deeper layers in our FWM.

3.3 α and diet of mixotrophic ciliates

The feeding habit of mixotrophic ciliates (FN 16-19) was relatively well known. They all showed kleptoplastidity (i.e. the retention of chloroplasts from a prey and their utilization for photosynthesis) and were considered as ‘obligate mixotrophic’, since they needed to engulf small microalgae to support their photosynthetic activity [12] (further details in Box 1). As already discussed above, mixotrophic oligotrichous

ciliates (FN 17-19) were introduced in the sole surface layer, while *M. rubra* population (FN 16) was widespread all over the water column. Thus, for each of these organisms, only one range of Max-Min α values was set.

In our FWM, we assumed that *Myrionecta rubra* (FN 16) behave mainly as a phototroph, being summer a favourite period for growth and cryptophytes always available in the GoN. The maximum ingestion rate reported for *M. rubra* accounted for 13.1 % of the ciliate body-mass per day at 18°C (recalculated data from [42]). Thus, a Max α of 0.131 d^{-1} was set, despite the difference of temperature between the experimental conditions at which it was measured (i.e. 18°C) and the temperature at which *M. rubra* was supposed to feed in the field in our model (25°C, surface layer, where cryptophyte showed the highest availability). In fact, a Q_{10} specific for *M. rubra* and other mixotrophic ciliates has never been established and we preferred to not use a generalist one, like that defined by [47] for ciliates. On the other hand, by adopting a Max α of 0.131 d^{-1}, we kept a convergence between ingestion rate and the fraction of metabolic requirements gained via heterotrophy (i.e. 10%, see former sections) in *M. rubra*. Finally, since this organism performed photosynthesis intensively, the Min α was assumed to be close to zero, considering the possibility of *M. rubra* to induce division in captured plastids.

Specific ingestion rate for *Strombidium rassoulzadeganii* approached 3 d^{-1} at concentrations of food comparable to those found in our system in the surface layer (<0.5 g C m^{-3}) but under a temperature of 19°C [44]. We set Max α for *Strombidium* spp. (FN 19) at 3 d^{-1}, despite temperature differences, for the same reason as above. Min α was set at 0.19 d^{-1}, since at saturating light (conditions at which the ingestion rate was at its minimum and photosynthesis at its maximum), the cell covered about the 81% of its metabolic need via photosynthesis (see Box 1). Concerning diet, the genus *Strombidium* performed kleptoplastidy at expenses of prasinophytes, cryptophytes, haptophytes and chlorophytes ([12] and refs therein), these groups being included in FN 11 and 12 in our model. Moreover, a ciliate with an ESD of about 30 µm, alike *Strombidium*, ate different kinds of particles, from bacteria to the largest ciliates [63]. Nonetheless, since microalgae in the above-mentioned classes were crucial for the ciliate to pursue its phototrophic metabolism, we assigned a relatively higher weight to them in FN 19 diet-matrix in our FWM.

Laboea strobila (FN 18) ingested up to 5.9% of its biomass per hour, giving a Max α of 1.42 d^{-1} [12], assuming that ingestion rates were equivalent during day and night (but this is not always true, see [64]). Min α was set at 0.36 d^{-1} by assumption, following the same rationale adopted for the other oligotrichous ciliates. Concerning diet, alike *Strombidium*, *L. strobila*, with an ESD of about 44 µm, ingested from bacteria to the largest ciliates. Yet, a higher ingestion of flagellate microalgae in FNs 11-12 was largely reasonable, for the reasons already discussed for *Strombidium*.

No information was available for the genus *Tontonia* (FN 17). The consumption rate was set at 3 d^{-1} by assumption - i.e. it was identical to that of *Strombidium*, for similarities of cellular biomass and metabolism (i.e. phototrophy/heterotrophy). Min α was set at 0.17 d^{-1}, according with the rationale above. Diet in FN 17 was identical to the other two oligotrichous plastidic ciliates.

3.4 α and diet of heterotrophic ciliates

Scanty information was available for Prostomatids (FN 22 and 36). The functional response for *Tiarina fusus* at 17°C was studied in the lab [46]. The highest specific ingestion rates were gathered when the species was fed with the dinoflagellate *Lingulodinium polyedrum*, a species with an ESD ~31 µm, while ingestion was considerably lower with smaller preys. *T. fusus* reached saturation for food concentrations above 1 gC m^{-3} (value never reached in the GoN during summer), while the weight-specific ingestion rate below this threshold was 0.21-2.1 d^{-1}. This range was taken as the Min-Max α range in the deep prostomatids in our model (i.e. *FN* 36). Considering a Q_{10} of 2.8 specific for ciliates ([47] and references therein), α for prostomatids in the surface layer (FN 22) was 0.77-4.7 d^{-1}.

Concerning diets of prostomatids, according to meta-analyses, prostomatids with an ESD of about 54 µm (as in FWM) could ingest all micro-plankters, from bacteria to ciliates [63]. We gave a relatively higher weight to trophic-links with food-items having an ESD > 12 µm (based on [46]). In a laboratory experiment, diatoms were not selected for by a prostomatid ciliate and a *Strobilidium* species [65], suggesting that these algae represent a low-quality food for ciliates. To be more neutral, due to the paucity of data, we let

prostomatids eat also diatoms in our FWM, although we imposed a relatively lower predatory pressure over them.

Concerning *Strobilidium* (FN 23 and 37, in surface and deeper layers, respectively), rates of weight-specific ingestion ranging 0.03-0.16 h^{-1} were reported in the literature for *Strobilidium* cf. *spiralis* fed different kinds of preys [66]: the higher value in the range was gained at saturation, corresponding to a concentration of food of 250 mgC m^{-3}, a value in the range of our system of study during summer. Assuming that ingestion rates at light and dark periods were comparable for *Strobilidium* (according to [64]), Max-Min α for *Strobilidium* in our model (FN 23) were set at, respectively, 3.84 and 0.72 d^{-1} (based on [66]). This range referred to a single temperature (i.e. 20°C, intermediate between our environmental extremes) but we considered it as representative for both surface and deeper layers populations (FNs 23 and 37). Concerning diet-matrix, being *Strobilidium* size comparable with that of prostomatids, the same prey-range was applied to it.

Concerning Tintinnids, ingestion rates at 25°C for *Tintinnopsis acuminata* ranged 0.73-7.3 d^{-1} (recalculated data from [67]), assuming that ingestion rates during day and night were comparable (no info available). These were our Max-Min α for Tintinnids in the surface layer (FN 24). Applying a Q_{10} of 2.8, α ranged 0.26-2.61 d^{-1} for Tintinnids in the deeper layer (FN 38). Diet of tintinnids was established based on the dimension of the tintinnid assemblage present in our system. The maximum particle size ingested by tintinnids was around 43% of the diameter of lorica at oral opening [68]. The average diameter for FN 24, weighted to each species relative abundance, was about 20 µm. The probable maximum size edible by tintinnids in our model was thus 9 µm. Moreover, in the laboratory, tintinnids eating phytoflagellates (FN 12 in our FWM) grew at rates similar to those eating diatoms lacking significant external processes (FN 7 in our FWM) [69]. Moderate growth was observed when tintinnids ate dinoflagellates of edible size (FN 14) while extensive mortality was reported in tintinnids fed with *Synechococcus* (FN 1) [69]. The diet of FN 24 and 38 was set based on considerations above.

Nanociliates (FNs 25 and 39, in surface and deeper layers, respectively) in our model included all ciliates with a cell-size < 20 µm (average ESD = 16 µm). The marine nanociliate *Uronema* (ESD about 12 µm, a size comparable to those in our model) ingested up to 31 *Synechococcus* cells ciliate^{-1} hour^{-1} at light conditions [70]. Since the ingestion rate by the nanociliates was 120% higher in dark than in light conditions (data for *Balanus comatum*, [64]), we estimated that the whole amount of cells ingested per day by one nanociliate was 68.2, giving a specific rate of ingestion of 1.89 d^{-1} (recalculation of original data). Since the experimental data considered were gained at 20°C (intermediate between 15 and 25°C) [70], we set 1.89 d^{-1} as the Max α for both the surface and deeper populations (FN 25 and 39). Since the functional response was unknown for nanociliates, the Min α at surface and deeper layers were assumed to be proportional to the Max α and the ratio between Min and Max α of other heterotrophic ciliates (i.e. Prostomatids and *Strobilidium*). The assumed Min α for FN 25 and 39 was 0.33 d^{-1}. Concerning diet of nanociliates, published meta-analyses indicated that they had been always tested in feeding experiments with cell-preys smaller than 9 micron ESD [63]. We were confident that that value represented an upper threshold-size for nanociliate feeding. Furthermore, the prostomatid *Uronema* spp. ate preferentially more *Synechococcus* than *Prochlorococcus* cells and heterotrophic bacteria [70].

3.5 α and diet of cladocerans

The feeding behaviour of *Penilia avirostris* (FN 42) from the Mediterranean Sea was studied in the laboratory and weight-specific ingestion rates ranged 0.26-1.57 d^{-1} at Summer conditions (natural photoperiod and temperature 22-27°C) [17]. A wider range for the weight-specific ingestion rate, i.e. 0-7 d^{-1}, was reported at about 22°C [18], with Min and Max values corresponding to food concentrations of about 0 and above 250 µg C L^{-1} (saturating condition). A value of 3.50 d^{-1} (recalculated data) was reported at about 203 µg C L^{-1}, i.e. the maximum potential food concentration in our model system (including the bacterial fraction too). Since mesozooplankton in our model was able to go up and down the seasonal thermoclyne, Max and Min α values for *P. avirostris* were chosen at the extremes of the natural food range in the two food-webs. Based on regression derived from the literature [18], Max-Min α ranged 0.71-3.5 d^{-1} (with *P. avirostris* food concentrations ranging 21-203 mg m^{-3}) at Green state, and 0.65-0.95 d^{-1} (with *P. avirostris* food concentrations ranging 17-37 mg m^{-3}) at Blue state.

Concerning diet, *P. avirostris* showed a lower prey-size of about 1 µm, corresponding to the ESD of the cyanobacterium *Synechococcus* ([17], grazing experiment using natural diet). The highest grazing pressure was exerted on flagellates, diatoms and dinoflagellates, while ciliates were grazed at lower rates due to their motility and larger size [17]. Most diatoms would be excluded from the preferred food of cladocerans, since long cell-colonies are not easily managed by these animals. Yet, we chose to consider all diatoms as a source of food to cladocerans based on the observation that these microalgae produced short or no chain in the GoN during summer (Sarno & Zingone personal communication). The diet-matrix for *Penilia* was set according to grazing-selectivity gained in experimental observations [18]. These reported: i) the highest grazing rates (selectivity index W' = 1) for food particles between 15 and 70 µm in size, corresponding to 6-28 micron µm and ii) no selection for bacterioplankton (neither auto- nor heterotrophic), although passive interception of these feeding particles was also possible. The grazing pressure on particles between 28 and 40 µm ESD and below 6 µm, excluding bacteria, was lower (W' =0.6-0.74). Conversely, in the range 40-60 µm ESD, selection was the lowest possible (W' = 0.25). Above 60 µm ESD, no feeding was detected.

The feeding performances for the other cladocerans in our model, including the genera *Evadne* and *Pseudoevadne* (FN 43), had been less investigated in comparison to *P. avirostris* and we set the ingestion rates for FN 43 equal to that set for FN 42. However, food-selectivity data for *Evadne normanni* suggested that selection by this species was operated on food particles different from those selected by *Penilia* [71]: the highest grazing pressure was exerted on particles with an ESD between 40 and 60 micron (W' = 1) and between 3 and 6 micron (W' = 0.76) (recalculated data).

3.6 α and diet of calanoid copepods

Feeding performances of copepods have been extensively studied at both in situ and laboratory conditions. By compiling data from several published papers, some authors conducted meta-analyses to sort for global relations between feeding rates and i) body-biomass, ii) amount of food available and iii) temperature [72,73]. It was suggested that, in this order, food concentration and body weight were the main drivers of changing of feeding rates of copepods in nature. Food concentration alone explained 52% of the variance in the (log) ingestion rates. Temperature had a limited influence of ingestion rates. Based on experimental and field data, multiple regression analysis were described to derive mathematically the ingestion rate from these driving factors [72,73]. We used these mathematical relations to find the most probable values of α for the main calanoid copepods in our model, i.e. *P. parvus*, *A. clausi*, *T. stylifera*, *C. typicus*, other calanoids and juvenile calanoids (respectively, FN 44-49). Each of these FNs were relatively small copepods, i.e. having a body-weight < 10 µg C ind^{-1}. Known variables in our calculations were i) FNs' body-biomass (see Table 1) and ii) the amount of carbon allocated into microbial FN with ESD ≥ 3 µm - i.e. the lowest threshold size for food particles available to copepods - at Green and Blue states and in the surface and deeper layers (i.e. 203-21 and 37-17 mgC m^{-3}, respectively). Data of feeding rates for copepods are shown in Dataset 1.

Calanoid copepods in our FWM ate wide range of prey, i.e. all unicellular organisms with the exception of prokaryotes. Nonetheless, selectivity was modulated differently for each copepod species according to the feeding preferences listed in the next paragraphs (Diet matrices in Dataset 2).

Paracalanus spp. (FN 44) collected small particles (< 5 µm, ESD 2 µm) only passively [74]; yet, in the laboratory, animals appeared to select for diatoms < 30 µm in size in a mixed-diatoms diet [20]. Information for *Paracalanus* feeding was anyway scanty and we assigned relatively higher preferences towards small microalgae and lower towards protozooplankton.

Prey of *Acartia clausi* (FN 45) ranged 7.5-250 µm in size [18]. Yet, selectivity was operated in the range of 70-100 µm in size, thus over mainly large diatoms, which were the most abundant food typology in the experimental observation considered herein [18]. It was thus possible that *A. clausi* was a peak tracker (i.e. it selected for the most abundant food). To overcome these uncertainties, we assigned low feeding selectivity to *A. clausi* in our model with the only exception of a extremely low pressure exerted over smaller organisms (ESD ~ 3 µm, i.e. nanoflagellates).

T. stylifera (FN 46) was reported to select for cells larger than those selected by *A. clausi* - i.e. particle retention efficiency was accounting for 100% for cell-ESD > 10 µm, and only ~25% for cell of 5 µm [75]. *T. stylifera* individuals from the GoN selected mainly cells and colonies ranging 30-200 µm [20]. In our model, the grazing pressure was established based on observations above: the highest pressure was on

particles exceeding 10 µm in ESD, moderate pressure between 7.5 and 10 µm, lower between 7.5 and 5 µm, and the lowest for ESD smaller than 5 but larger than 2 µm.

Laboratory studies have reported *C. typicus* (FN 47) feeding upon a wide size-range of organisms including small algae [76], Appendicularia juveniles and eggs [77] and juvenile copepods [78]. The optimal prey-size for *C. typicus* was represented by particles >10 µm, but regarding the upper size limit, this copepod ingested prey larger than its own size, like yolk-sac fish larvae ([79] and references therein). Differently from *T. stylifera*, which was reported as a cruiser/suspension feeder, *C. typicus* was capable also of ambush predation and selected for large motile preys, such as ciliates or dinoflagellates, both in nature and laboratory [79]. In our model, we gave i) the highest selectivity rank to motile preys exceeding 10 µm in ESD, ii) an intermediate value to non-motile preys above 10 µm and iii) the lowest rank to those below this threshold.

The diet of other calanoids (FN 48), dominated by the small copepods *Clausocalanus* spp., was assumed similar to that of *P. parvus* for taxonomical affinity between the latter and the copepods species included in FN 48 (i.e. small copepods in the genera *Clausocalanus* and *Paracalanus*).

Trophic behavior of copepodites of calanoid copepods (FN 49, including mainly *Paracalanus* and *Clausocalanus*) was set similar to that of adults of *Paracalanus*.

3.7 α and diet of **Oithona** *spp.*

The cyclopoid copepods *Oithona* spp. (FN 54, including copepodites and adults of different species) showed a feeding behaviour distinct from other copepods. The definition of ingestion rates of *Oithona* spp. was based on data for the species *O. davisae* at different life stages [80,81]. The Max-Min values of α where estimated based on regressions derived from published studies [80,81] and at the four key-concentrations considered in our model (see above).

Concerning diet of *Oithona* spp., according to [82], faecal pellets provided the 20-30% of daily ratio of these animals, but *Oithona* spp. ate also copepod juveniles, so that carnivorous feeding accounted for at least 25% of the daily ratio [83]. It was also reported that ciliates accounted for at least the 25% of the daily up-taken carbon and that these protists were anyway preferred within the microplankton [84]. Moreover, ciliates and dinoflagellates represented up to 80% of the diet of *O. similis* [85]. Concerning phytoplankton, a lower food-size limit of 10 µm was reported [86]. To this respect, we assumed that the diet of *Oithona* included 40% of ciliates and dinoflagellates, 25% of detritus (as faecal pellets), 25% of juvenile copepods and 10% of phytoplankton (size > 10 µm).

3.8 α and diet of Appendicularia

The bioenergetics of the appendicularian *Oikopleura dioica* was described in detail in laboratory conditions (T=15°C) [28,29]. The Max-Min α in our model for Appendicularia (FN 50) were defined based on published regressions [28](Table 2 in that paper), by relating α to the body-weight of these animals in our model and the four key food-concentrations at the two system states (see above). Values for surface layers have been increased according to a Q_{10} of 1.78 specific for grazing rates of *O. dioica* [57].

Being filter feeders, Appendicularia ingested all particulate material, from bacteria to microzooplankton, and even small detritus particles. In our model, we excluded detritus from the spectrum of food available to these organisms for some reasons: firstly, in surface waters, during the physically stable summer season and in the course of phytoplankton matter production and retention in the mixed layer, aggregates exceeding the size of appendicularians considered in the model (~ 1 mm) are more frequently developed; secondly, in the deeper layer, a large fraction of detritus would be composed by large and heavy faecal pellets sinking to the bottom. The weight-specific clearance of *Oikopleura* spp. was put in relation with the size of food particles: this rate showed the highest values for particles with an ESD between 20 and 60 µm [87]. Above this range, the clearance was 10 times lower; below it, the rate was 100-10 times lower than in the range mentioned above. In line with these results, weight-specific ingestion of eukaryotes with ESD <13 µm was reported almost three times higher than that of prokaryotes [88]. Diet of FN 50 was arranged accordingly.

3.9 α and diet of doliolids and salps

The functional response (ingestion vs. food concentrations) was described in detail for *Doliolum denticulatum* from the North Western Mediterranean in individuals cultivated in laboratory conditions (T ~22°C) and fed with natural microplankton community [18]. These animals had a size comparable to that of *D. nationalis*, the doliolid species most frequently recorded in the GoN. The α rates of Doliolids in our FWM (FN 51) were estimated based on regressions derived from the study above at the four key concentrations in our model.

Doliolids' diet included food-particle within a wide range of size, from bacteria to microplankton. A higher selectivity was reported for particles between 2.5-7.5 µm in size (Selectivity index W' = 1) [18]. Nonetheless, relatively high indexes were also reported for slightly smaller and larger particles (W' = 0.99 and 0.94 for particles around 2.5 µm and ranging 7.5-15 µm in size, respectively), while particles larger than 100 µm were not grazed. Selectivity indexes for picoplankton (including bacteria) and for microplankton between 15 and 100 µm were around half the maximum mentioned above, thus relevant. Since in our model only a few tintinnid species exceeded 100 µm in the longest linear dimension, we included all pico-, nano- and microplankton in Doliolids' diet in our FWM, giving different weights to the different trophic links based on the considerations above.

Ingestion rates of Salps (FN 52) have been less easily investigated than those of Doliolids, since, in comparison with the latter, salps are bigger organisms hardly kept alive at laboratory conditions. Salps with a size < 10 mm (the dimensional category more easily detected in mesozooplankton samples at the coastal station MC) showed weight-specific ingestion rates ranging 2-8.3 % of the body carbon per hour (α ~ 0.24-1 d^{-1}, assuming that animal ate 12 hours a day) ([36] and reference therein). These values were in the range of those gained in nature for salps at extreme trophic conditions - bloom vs. non bloom conditions in the Southern Ocean - (i.e. 0.1-1 d^{-1}, respectively, [89]), thus at temperature <15°C. As for salps living at higher temperature (like in the Gulf of Naples), assuming a Q_{10} of 1.78, the Max α for both Green and Blue conditions was 1.78 d^{-1}. In our model, we assumed that α ranged 0.24-1.78 d^{-1} for salps, thus enough to sustain the potentially high µ rates assumed for these organisms (see section 2.6). These values were also in the range of those gained in other studies on salps (e.g. [90,91]). Moreover, based on data regressions [91], saturation of salps' filter-feeding was gained at about 10 µgC L^{-1}. Since in our system of study the concentration of food available to salps always exceeded that threshold, we adopted the same value of Max α for both Green and Blue conditions.

Salps are non-selective feeders, eating from bacteria to the largest microplankton and small detritus-aggregates. In our model, we did not included detritus in the diet of salps for the same reason we did for doliolids. In the course of a feeding experiment with microspheres, a high affinity for particles with size < 3 µm, i.e. bacteria, virus and colloids, was reported for the gut of salps, suggesting that these could be an important source of food at low microplankton concentration [92]. In the experiment mentioned above, about the 60 % of particles in the gut of the tested salps were within 1 µm in size at concentrations in the order of 10^3 cell ml^{-1}, thus comparable to those found in our system at any state and depth of the water column. According to the authors of that study, even though flagellates and small diatoms (particles > 1 µm) represented a larger carbon-pool in nature due to their abundance, prokaryotes and smaller particles (in the range 0.1-1 µm) were more quickly digestible because they presented a relatively higher surface area and they could fully satisfy salp energetic needs. Although these observations were not easy to be generalized for our modelling purposes - since they did not considered particles larger than 3 µm (the majority in our model) - they supported the view on possible selective feeding in salps. Thus, we included in our model the constraint that the 60% of the daily ration of Salps (FN 52) came from bacteria, cyanobacteria and prochlorophytes.

3.10 α and diets of meroplankton, detritivores and carnivores

Feeding rates of meroplanktonic larvae (FN 53) typically found during summer in the GoN and belonging to Polychaeta, Echinoidea, Cirripeda, Bivalvia and Gastropoda were measured over natural microplankton communities and a specific ingestion rate of 2.5 d^{-1} was reported for each of these groups [31]. These rates referred to a natural phytoplankton-bloom in the Vancouver bay during July with temperature ranging 13-17°C and at relatively high food concentration – estimated via chlorophyll concentration, ranging 2-6 µg L^{-1} (thus assimilated to Green conditions in the Gulf of Naples in surface waters). Assuming Q_{10}=2.8 in general

for marine planktonic animals [47], the Max α during summer in the GoN accounted for 7 d^{-1}, which was a very high rate. Yet, meroplanktonic larvae have high energetic requirements to pursue an intense somatic growth and such high rates were not improbable. Being information on functional response of meroplankton virtually absent in the literature, the minimum rate was set as proportional to the lowest Chl a concentration in our model-system: i.e. Min α = Max α * 0.1 = 0.7 d^{-1}, where 0.1 is the ratio between Min and Max Chl a concentrations in our model system (see previous sections). An identical α range was assumed for both Green and Blue conditions.

Concerning meroplankton diet, meroplankton ate different kinds of particles, from nanoflagellates (both photo- and heterotrophic), diatoms, dinoflagellates (of different sizes) and ciliates [31]. According to that study, the highest pressure was exerted on the most abundant species, the harmful photosynthetic flagellate *Heterosigma akashiwo* (Raphidophycea, in our model, assimilated to algae in FN 12), followed by dinoflagellates, nanoflagellates and ciliates, while diatoms were weakly impacted by meroplankton grazing. We must stress here the fact that the above-mentioned study referred to a so-called 'harmful algal bloom condition', with *H. agashiwo* abundance largely exceeding that of diatoms (*H. agashiwo* was ten times more abundant than all diatoms taken collectively). Thus, it was not improbable that meroplanktonic larvae behave as peak-trackers at specific trophic conditions. In our model, due to the apparently controversial information of the trophic behavior of meroplanktonic larvae, these animal were enabled to eat all unicellular plankton, with the exception of prokaryotes.

Detritivores (FN 55) in our model includes only cyclopoid and harpacticoid copepods feeding specifically on Appendicularia houses. Among them, *Oncea mediterranea* ate up to 100% its body-biomass, at 20°C [93]. According to the regression described by [72], Max α for *FN* 55, was 1.23 d^{-1}, considering its body-size. The Min α was ~0.1 d^{-1}, a value similar to the minimum ingestion rate of other copepods.

Carnivores in our model (FN 56) included chaetognaths, pteropods, siphonophors and some strictly carnivore copepods (*Candacia* spp. and *Pleuromamma* spp.). Chaetognats were the most abundant group, with pteropods, the second in rank, being about one fifth of the former. In the model, we assumed that the ingestion rate of the carnivore FN was mainly driven by chaetognats' behaviour. The feeding performances (by analyzing the gut content) for different *Sagitta* species were studied in detail at different maturity stages and across a coast-offshore trophic gradient in the Western Mediterranean during summer: ingestion rates ranged 0.13-3.76 preys ingested per chaetognath per day and no relation with food concentration was detected [94]. Relating to our model, considering an average weight of 18.35 µg C per animal for the FNs which were potentially eaten by chaetognaths, α ranged 0.013-0.37 d^{-1}, at both Green and Blue states. Among the identified preys in the animal gut, copepods were the most abundant ones, while cladocerans were one tenth less [94]. Among copepods, relatively larger species (i.e. *C. typicus*, *T. stylifera*, *Candacia* spp., *Pleuromamma* spp.) were more intensively caught than smaller ones (*Paracalanus* spp., *Acartia* spp., *Clausocalanus* spp.). Other crustaceans and chaetognats were also present in the diet, but at lower abundance. The diet of FN 56 in our model was set according to the food preferences listed above.

4. Derivation of Not-Assimilated fraction of Consumed Biomass (ε)

The parameter ε is the complement to 1 of the assimilation efficiency (AE), i.e. the amount of the food consumed that is destined to respiration and growth (i.e. assimilated). AE was here considered the better proxy for defining the Ecopath parameter ε, i.e. the not-assimilated fraction of consumed biomass.

The assimilation efficiency of mixo- and heterotrophic unicellular organisms in our model was assumed ~1, according to [12], and ε was set at 0.01.

The AE of cladocerans (FN 42-43) was in the range of 0.70-0.45, respectively, from the lowest to higher food concentrations, within the range of variability of our study system [18,71], giving ε ranging 0.55-0.30 for both Green and the Blue states.

AE of calanoid copepods (FN 44-48) was set based on information available for *A. clausi* [18]: under different food concentrations, that copepod showed AE ranging 0.78-0.45, within a wide range of food concentrations, i.e. from ~0 to over 800 µg C L^{-1}. Nevertheless, the regression of AE vs. food concentration was not statistically significant [18], since some values were comprised between 0.6 and 0.8 at super-saturating food concentrations, opening to the possibility that assimilation could be more efficient at some

conditions. In the range of food variability of our study system, and considering a linear regression fitting data-points, AE ranged ~0.68-0.40, with a consequent ε ranging 0.32-0.60, for both Green and Blue states. This ε range was assumed to be extensible to all calanoid copepods in our model (FN 44-48), excepting juvenile stages (FN 49), whose efficiency was set at a fixed value of 0.73, assuming that it was equal to that of *Oithona davisae* juveniles at 20°C [95]. Such a high value was related to higher somatic growth shown by juvenile copepod (absent in adults). ε of juvenile calanoids was thus fixed at 0.27.

AE of Appendicularia (FN 50) was inversely related to food concentration [28,29]. The latter authors described a regression linking AE to food concentration, used herein to infer AE values for our concentration and temperature extremes (recalculated data, considering also a Q_{10} of 1.78 as reported by [57]. In our model system, ε ranged 0.091-0.604 in the Blue state, and 0.091-0.945 in the Green state. The wide range of AE for Appendicularia (and also other gelatinous mesozooplankton) stemmed from the higher influence exerted by external conditions in metabolic processes (as feeding) in these organisms, if compared with copepods.

AE of Doliolids (FN 51) ranged 0.72-0.32 in the range of food of our study system, with a slight negative relation to food concentration for values of the latter variable exceeding 400 mg C m^{-3} [18]. The calculated ε for our system using the regression described by the above-mentioned study ranged 0.25-0.58 and 0.28-0.68 in Blue and Green conditions, respectively.

AE of Salps (FN 52) within 10 mm in length (as in our model) would account for 67 % [96], but this could be only a value in a range and homologous data are very scanty in the literature. However, comparing to the other filter feeders, salps' AE seems to be relatively less dependent from food concentration. Salps of 10 mm showed AE comparable to smaller ones, ranging 75-87% of the body carbon-biomass at two different states of carbon concentration and microplankton composition during an iron fertilization experiment [97]. We can thus suppose that the assimilation efficiency is quite stable under different food concentrations, since salps can also eliminate water and food in excess by means of backwashing, i.e. expelling water in excess and regulate feeding [36]. ε of Salps in our model (FN 52) was set as ranging 0.13-0.25 at both Green and Blue states.

AE ranging 65-86% for different life stages was estimated for the cyclopoid copepod *Oithona* (FN 54) [95]. No other data were available for this copepod and we assumed that ε ranged 0.24-0.35 at both Green and Blue states. Considering that *Oithona* spp. were ambush feeders, it was probable that saturation emerged at concentrations higher than those critical for suspension feeders, thus giving a narrow range of variability for AE ε.

Information about AE for meroplankton (FN 53) were not available. We assumed that ε was 0.2 in this group at both Green and Blue states. This was a relatively high value, but it was plausible since larvae should convert in somatic growth a large part of the matter ingested.

Assimilation efficiency of detritivora (FN 55) - a minor group of mesozooplankton composed mainly by cyclopod and harpacticoid copepods - was assumed equal to that of *Oithona*. Being detritivors opportunistic feeders, similarly to *Oithona*, this assumption is highly plausible.

Carnivorous mesozooplankton (FN 56), mainly chaetognats in our model, were reported to have a high AE, i.e. ranging 80-82% [98,99]. These rates were similar to other strictly carnivorous plankton (e.g. siphonophors, a minor sub-group of animals in our model ([35]). ε in FN 56 ranged 0.18-0.2, at both Green and Blue states.

Supplementary References

1. D'Alelio, D. *et al.* The green – blue swing : plasticity of plankton food-webs in response to coastal oceanographic dynamics. *Mar. Ecol.* doi:10.1111/maec.12211
2. Kana, T. M. & Glibert, P. M. Effect of irradiances up to 2000 μE m-2 s -l on marine Synechococcus WH7803--I. Growth, pigmentation, and cell composition. *Deep. Res.* **34,** 479–495 (1987).

3. Campbell, L., Nolla, H. A. & Vaulot, D. The importance of Prochlorococcus central North Pacific Ocean to community structure in the central North Pacific Ocean. *Limnol. Oceanogr.* **39,** 954–961 (1994).

4. Menden-Deuer, S. & Lessard, E. J. Carbon to volume relationships for dinoflagellates, diatoms, and other protist plankton. *Limnol. Oceanogr.* **45,** 569–579 (2000).

5. Modigh, M., Saggiomo, V. & Ribera d'Alcalà, M. Conservative features of picoplankton in a Mediterranean eutrophic area , the Bay of Naples. **18,** 87–95 (1996).

6. Casotti, R., Brunet, C., Aronne, B. & Ribera d'Alcalà, M. Mesoscale features of phytoplankton and planktonic bacteria in a coastal area as induced by external water masses. *Mar. Ecol. Prog. Ser.* **195,** (2000).

7. Jeong, H. J. *et al.* Growth, feeding and ecological roles of the mixotrophic and heterotrophic dinoflagellates in marine planktonic food webs. *Ocean Sci. J.* **45,** 65–91 (2010).

8. Crawford, D. W. Mesodinium rubrum: the phytoplankter that wasn't. *Mar. Ecol. Prog. Ser.* **58,** 161–174 (1989).

9. Johnson, M. & Stoecker, D. Role of feeding in growth and photophysiology of Myrionecta rubra. *Aquat. Microb. Ecol.* **39,** 303–312 (2005).

10. Johnson, M. D., Stoecker, D. K. & Marshall, H. G. Seasonal dynamics of Mesodinium rubrum in Chesapeake Bay. *J. Plankton Res.* **35,** 877–893 (2013).

11. Gustafson, D. E., Stoecker, D. K., Johnson, M. D., Van Heukelem, W. F. & Sneider, K. Cryptophyte algae are robbed of their organelles by the marine ciliate Mesodinium rubrum. *Nature* **405,** 1049–1052 (2000).

12. Stoecker, D., Johnson, M., deVargas, C. & Not, F. Acquired phototrophy in aquatic protists. *Aquat. Microb. Ecol.* **57,** 279–310 (2009).

13. Dolan, J. R. & Pérez, M. T. Costs , benefits and characteristics of mixotrophy in marine oligotrichs. *Freshw. Biol.* **45,** 227–238 (2000).

14. Ribera d'Alcalà, M. *et al.* Seasonal patterns in plankton communities in a pluriannual time series at a coastal Mediterranean site (Gulf of Naples): an attempt to discern recurrences and trends. *Sci. Mar.* **68,** 65–83 (2004).

15. Zingone, A., Montresor, M. & Marino, D. Summer phytoplankton physiognomy in coastal water of the Gulf of Naples. *Mar. Ecol.* **11,** (1990).

16. Lee, S. & Fuhrman, J. E. D. A. Relationships between Biovolume and Biomass of Naturally Derived Marine Bacterioplanktont. *Appl. Environ. Microbiol.* **53,** 1298–1303 (1987).

17. Atienza, D., Saiz, E. & Calbet, a. Feeding ecology of the marine cladoceran Penilia avirostris: natural diet, prey selectivity and daily ration. *Mar. Ecol. Prog. Ser.* **315,** 211–220 (2006).

18. Katechakis, A., Stibor, H., Sommer, U. & Hansen, T. Feeding selectivities and food niche separation of Acartia clausi, Penilia avirostris (Crustacea) and Doliolum denticulatum (Thaliacea) in Blanes Bay (Catalan Sea, NW Mediterranean). *J. Plankton Res.* **26,** 589–603 (2004).

19. Walve, J. & Larsson, U. Carbon , nitrogen and phosphorus stoichiometry of crustacean zooplankton in the Baltic Sea : implications for nutrient recycling. *J. Plankton Res.* **21,** 2309–2321 (1999).

20. Mahadik, G. A. The role of copepod grazing in phytoplankton bloom dynamics: a species-based approach. (2014).

21. Halsband-Lenk, C., Nival, S., Carlotti, F. & Hirche, H.-J. Seasonal cycles of egg production of two planktonic copepods , Centropages typicus and Temora stylifera , in the north-western Mediterranean Sea. *J. Plankton Res.* **23,** 597–609 (2001).

22. Nikolioudakis, N., Isari, S., Pitta, P. & Somarakis, S. Diet of sardine Sardina pilchardus: an 'end-to-end' field study. *Mar. Ecol. Prog. Ser.* **453,** 173–188 (2012).

23. Nassogne, A. Etudes préliminaires du zooplankton dans la constitution et le transfert de la matière organique au sein de la chaîne alimentaire marine en Mer Ligure. Radioattiva del mare. (1972).

24. Mazzocchi, M. G. & Paffenhöfer, G.-A. First observations on the biology of Clausocalanus furcatus (Copepoda , Calanoida). *J. Plankton Res.* **20,** 331–342 (1998).

25. Sato, R., Ishibashi, Y., Tanaka, Y., Ishimaru, T. & Dagg, M. J. Productivity and grazing impact of Oikopleura dioica (Tunicata, Appendicularia) in Tokyo Bay. *J. Plankton Res.* **30,** 299–309 (2007).

26. Sato, R., Tanaka, Y. & Ishimaru, T. House Production by Oikopleura dioica (Tunicata, Appendicularia) Under Laboratory Conditions. *J. Plankton Res.* **23,** 415–423 (2001).

27. Acũna, J. L. & Kiefer, M. Functional response of the appendicularian Oikopleura dioica. *Limnol. Oceanogr.* **45,** 608–618 (2000).

28. Lombard, F., Renaud, F., Sainsbury, C., Sciandra, A. & Gorsky, G. Appendicularian ecophysiology I Food concentration dependent clearance rate, assimilation efficiency, growth and reproduction of Oikopleura dioica. *J. Mar. Syst.* **78,** 606–616 (2009).

29. Lombard, F., Sciandra, A. & Gorsky, G. Appendicularian ecophysiology. II. Modeling nutrition, metabolism, growth and reproduction of the appendicularian Oikopleura dioica. *J. Mar. Syst.* **78,** 617–629 (2009).

30. Heron, A. C. Length-weight relation in the salp Thalia democratica and potential of salps as a source of food. *Mar. Ecol. Prog. Ser.* **42,** 125–132 (1988).

31. Almeda, R., Messmer, A. M., Sampedro, N. & Gosselin, L. A. Feeding rates and abundance of marine invertebrate planktonic larvae under harmful algal bloom conditions off Vancouver Island. *Harmful Algae* **10,** 194–206 (2011).

32. Gorsky, G., Dallot, S., Sardou, J., Fenaux, R. & Palazzoli, I. C and N composition of some northwestern Mediterranean zooplankton and micronekton species. *J. Exp. Mar. Bio. Ecol.* **124,** 133–144 (1988).

33. Saito, H. & Kiørboe, T. Feeding rates in the chaetognath Sagitta elegans : effects of prey size , prey swimming behaviour and small-scale turbulence. (2001).

34. Lindley, J. a., John, A. W. G. & Robins, D. B. Dry Weight, Carbon and Nitrogen Content of Some Calanoid Copepods from the Seas Around Southern Britain in Winter. *J. Mar. Biol. Assoc. United Kingdom* **77,** 249 (1997).

35. Purcell, J. E. & Kremer, P. Feeding and metabolism of the siphonophore Sphaeronectes gracilis. *J. Plankton Res.* **5,** 95–106 (1983).

36. Bone, Q. & others. *The biology of pelagic tunicates*. (Oxford University Press Oxford, 1998).

37. Dilling, L. & Alldredge, A. L. Can chaetognath fecal pellets contribute significantly to carbon flux ? *Mar. Ecol. Prog. Ser.* **92,** 51–58 (1993).

38. Baines, S. B. & Pace, M. L. The production of dissolved organic matter by phytoplankton and its importance to bacteria: petterns across marine and freshwater systems. *Limnol. Oceanogr.* **36,** 1078–1090 (1991).

39. Sosik, H. M., Olson, R. J., Neubert, M. G., Shalapyonok, A. & Solow, A. R. Growth rates of coastal phytoplankton from time-series measurements with a submersible flow cytometer. *Limnol. Oceanogr.* **48,** 1756–1765 (2003).

40. Liu, H., Nolla, H. A. & Campbell, L. Prochlorococcus growth rate and contribution to primary production in the equatorial and subtropical North Pacific Ocean. *Aquat. Microb. Ecol.* **12,** 39–47 (1997).

41. Edwards, K. F., Thomas, M. K., Klausmeier, C. a. & Litchman, E. Allometric scaling and taxonomic variation in nutrient utilization traits and maximum growth rate of phytoplankton. *Limnol. Oceanogr.* **57,** 554–566 (2012).

42. Yih, W., Kim, H., Jeong, H., Myung, G. & Kim, Y. Ingestion of cryptophyte cells by the marine photosynthetic ciliate Mesodinium rubrum. *Aquat. Microb. Ecol.* **36,** 165–170 (2004).

43. Pérez, M. T., Dolan, J. R. & Fukai, E. Planktonic oligotrich ciliates in the NW Mediterranean: growth rates and consumption by copepods. *Mar. Ecol. Prog. Ser.* **155,** 89–101 (1997).

44. Schoener, D. & McManus, G. Plastid retention, use, and replacement in a kleptoplastidic ciliate. *Aquat. Microb. Ecol.* **67,** 177–187 (2012).

45. Weisse, T. Growth and production of heterotrophic nanoflagellates in a meso-eutrophic lake. *J. Plankton Res.* **19,** 703–722 (1997).

46. Jeong, H., Yoon, J., Kim, J., Yoo, Y. & Seong, K. Growth and grazing rates of the prostomatid ciliate Tiarina fusus on red-tide and toxic algae. *Aquat. Microb. Ecol.* **28,** 289–297 (2002).

47. Hansen, P. J. & Bjørnsen, P. K. Zooplankton grazing and growth : Scaling within the 2-2 ,000- µm body size range. *Limnol. Oceanogr.* **42,** 687–704 (1997).

48. Verity, P. G. Growth rates of natural tintinnid populations in Narragansett Bay. *Mar. Ecol. Prog. Ser.* **29,** 117–126 (1986).

49. Franzé, G. & Modigh, M. Experimental evidence for internal predation in microzooplankton communities. *Mar. Biol.* **160,** 3103–3112 (2013).

50. Atienza, D., Calbet, A., Saiz, E. & Lopes, R. M. Ecological success of the cladoceran Penilia avirostris in the marine environment: feeding performance, gross growth efficiencies and life history. *Mar. Biol.* **151,** 1385–1396 (2007).

51. Atienza, D., Saiz, E., Skovgaard, a., Trepat, I. & Calbet, a. Life history and population dynamics of the marine cladoceran Penilia avirostris (Branchiopoda: Cladocera) in the Catalan Sea (NW Mediterranean). *J. Plankton Res.* **30,** 345–357 (2008).

52. Egloff, D. A., Fofonoff, P. W. & Onbé, T. Reproductive biology of marine cladocerans. *Adv. Mar. Biol.* **31,** 80–167 (1997).

53. Frangoulis, C., Carlotti, F., Eisenhauer, L. & Zervoudaki, S. Converting copepod vital rates into units appropriate for biogeochemical models. *Prog. Oceanogr.* **84,** 43–51 (2010).

54. Hirst, a. G. & Bunker, a. J. Growth of marine planktonic copepods: Global rates and patterns in relation to chlorophyll a, temperature, and body weight. *Limnol. Oceanogr.* **48,** 1988–2010 (2003).

55. Deibel, D. & Lowen, B. A review of the life cycles and life-history adaptations of pelagic tunicates to environmental conditions. *ICES J. Mar. Sci.* **69,** 358–369 (2012).

56. Gibson, D. M. & Paffenhöfer, G. Feeding and growth rates of the doliolid , Dolioletta gegenbauri Uljanin (Tunicata , Thaliacea). *J. Plankton Res.* **22,** 1485–1500 (2000).

57. Broms, F. & Tiselius, P. Effects of temperature and body size on the clearance rate of Oikopleura dioica. *J. Plankton Res.* **25,** 573–577 (2003).

58. Almeda, R. *et al.* Feeding and growth kinetics of the planktotrophic larvae of the spionid polychaete Polydora ciliata (Johnston). *J. Exp. Mar. Bio. Ecol.* **382,** 61–68 (2009).

59. Novarino, G., Oliva, E. & Perez-Uz, B. Nanoplankton protists from the western Mediterranean Sea. I. Occurrence, ultrastructure, taxonomy and ecological role of the mixotrophic flagellate Ollicola vangoorii (Chrysomonadidae = Chrysophyceae p.p.). *Sci. Mar.* **66,** 233–247 (2002).

60. Callieri, C., Karjalainen, S. M. & Passoni, S. Grazing by ciliates and heterotrophic nanoflagellates on picocyanobacteria in Lago Maggiore , Italy. *J. Plankton Res.* **24,** 785–796 (2002).

61. Christaki, U., Giannakourou, A., Wambeke, F. V. A. N. & Grégori, G. Nanoflagellate predation on auto- and heterotrophic picoplankton in the oligotrophic Mediterranean Sea. *J. Plankton Res.* (2001).

62. Christaki, U., Vázquez-Domínguez, E., Courties, C. & Lebaron, P. Grazing impact of different heterotrophic nanoflagellates on eukaryotic (Ostreococcus tauri) and prokaryotic picoautotrophs (Prochlorococcus and Synechococcus). *Environ. Microbiol.* **7,** 1200–10 (2005).

63. Sailley, S. F. & Buitenhuis, E. T. Microzooplankton functional responses in the lab and in the field. *Earth Syst. Sci. Data Discuss.* **7,** 49–167 (2014).

64. Jakobsen, H. H. & Strom, S. L. Circadian cycles in growth and feeding rates of heterotrophic protist plankton. *Limnol. Oceanogr.* **49,** 1915–1922 (2004).

65. Müller, H. & Schlegel, A. Responses of three freshwater danktonic ciliates with different feeding modes to cryptophyte and diatom prey. *Aquat. Microb. Ecol.* **17,** 49–60 (1999).

66. Verity, P. G. Measurement and simulation of prey uptake by marine planktonic ciliates fed plastidic and aplastidic nanoplankton. *Limnol. Oceanogr.* **36,** 729–749 (1991).

67. Verity, P. G. Grazing , respiration , excretion , and growth rates of tintinnids. *Limnol. Oceanogr.* **30,** 1268–1282 (1985).

68. Heinbokel, J. F. Studies on the functional role of tintinnids in the Southern California Bight. II. Grazing rates of field populations. *Mar. Biol.* **47,** 191–197 (1978).

69. Verity, P. G. & Villareal, T. A. The Relative Food Value of Diatoms, Dinoflagellates, Flagellates, and Cyanobacteria for Tintinnid Ciliates. *Arch. für Protistenkd.* **131,** 71–84 (1986).

70. Christaki, U., Jacquet, S., Dolan, J. R., Vaulot, D. & Rassoulzadegan, F. Growth and grazing on Prochlorococcus and Synechococcus by two marine ciliates. *Limnol. Oceanogr.* **44,** 52–61 (1999).

71. Katechakis, A. & Stibor, H. Feeding selectivities of the marine cladocerans Penilia avirostris, Podon intermedius and Evadne nordmanni. *Mar. Biol.* **145,** 529–539 (2004).

72. Saiz, E. & Calbet, A. Scaling of feeding in marine calanoid copepods. *Limnol. Oceanogr.* **52,** 668–675 (2007).

73. Saiz, E. & Calbet, A. Copepod feeding in the ocean: scaling patterns, composition of their diet and the bias of estimates due to microzooplankton grazing during incubations. *Hydrobiologia* **666,** 181–196 (2011).

74. Price, H. J., Paffenhöfer, G.-A. & Strickler, J. R. Modes of cell capture in calanoid copepods. *Limnol. Oceanogr.* **28,** 116–123 (1983).

75. Dam, H. Short-term feeding of Temora longicornis Müller in the laboratory and the field. *J. Exp. Mar. Bio. Ecol.* **99,** 149–161 (1986).

76. Tomasini, J. A. & Mazza, J. Feeding behaviour of Centropages typicus (Copepoda, Calanoida) in nutritive media containing two algae, and in the presence of nauplii of Artemia sp. *J. Du Cons.* **38,** (1979).

77. López-Urrutia, A., Harris, R. P. & Smith, T. Predation by calanoid copepods on the appendicularian Oikopleura dioica. *Limnol. Oceanogr.* **49,** 303–307 (2004).

78. Titelman, J. Swimming and escape behavior of copepod nauplii: implications for predator-prey interactions among copepods. *Mar. Ecol. Prog. Ser.* **213,** 203–213 (2001).

79. Calbet, A., Carlotti, F. & Gaudy, R. The feeding ecology of the copepod Centropages typicus (Kröyer). *Prog. Oceanogr.* **72,** 137–150 (2007).

80. Almeda, R., Augustin, C. B., Alcaraz, M., Calbet, A. & Saiz, E. Feeding rates and gross growth efficiencies of larval developmental stages of Oithona davisae (Copepoda, Cyclopoida). *J. Exp. Mar. Bio. Ecol.* **387,** 24–35 (2010).

81. Saiz, E., Calbet, A., Broglio, E. & Marı, P. Effects of small-scale turbulence on copepods : The case of Oithona davisae. *Limnol. Oceanogr.* **48,** 1304–1311 (2003).

82. Gonzalez, H. E. & Smetacek, V. The possible role of the cyclopoid copepod Oithona in retarding vertical flux of zooplankton faecal material. *Mar. Ecol. Prog. Ser.* **113,** 233–246 (1994).

83. Lampitt, R. S. Carnivorous feeding by a small marine copepod. *Limnol. Oceanogr.* **23,** 1228–1231 (1978).

84. Castellani, C., Irigoien, X., Harris, R. & Lampitt, R. Feeding and egg production of Oithona similis in the North Atlantic. *Mar. Ecol. Prog. Ser.* **288,** 173–182 (2005).

85. Castellani, C., Irigoien, X., Mayor, D. J., Harris, R. P. & Wilson, D. Feeding of Calanus finmarchicus and Oithona similis on the microplankton assemblage in the Irminger Sea, North Atlantic. *J. Plankton Res.* **30,** 1095–1116 (2008).

86. Drits, A. V & Semenova, T. N. Experimental investigations of the feeding of Oithona similis Claus. *Oceanology* **24,** 755–759 (1984).

87. Vargas, C. & González, H. Plankton community structure and carbon cycling in a coastal upwelling system. I. Bacteria, microprotozoans and phytoplankton in the diet of copepods and appendicularians. *Aquat. Microb. Ecol.* **34,** 151–164 (2004).

88. Scheinberg, R., Landry, M. & Calbet, a. Grazing of two common appendicularians on the natural prey assemblage of a tropical coastal ecosystem. *Mar. Ecol. Prog. Ser.* **294,** 201–212 (2005).

89. Von Harbou, L. *et al.* Salps in the Lazarev Sea, Southern Ocean: I. Feeding dynamics. *Mar. Biol.* **158,** 2009–2026 (2011).

90. Deibel, D. Laboratory-measured grazing and ingestion rates of the salp Thalia democratica Forskal and the doliolid Dolioletta gegenbauri Uljanin (Tunicata , Thaliacea). *J. Plankton Res.* **4,** 189–201 (1982).

91. Andersen, V. Filtration and ingestion rates of Salpa fusiformis Cuvier (Tunicata, Thaliacea): Effects of size, individual weight and algal concentration. *J. Exp. Mar. Bio. Ecol.* **87,** 13–29 (1985).

92. Sutherland, K. R., Madin, L. P. & Stocker, R. Filtration of submicrometer particles by pelagic tunicates. *Proc. Natl. Acad. Sci. U. S. A.* **107,** 15129–34 (2010).

93. Paffenhofer, G. On the ecology of marine cyclopoid copepods (Crustacea , Copepoda). **15,** 37–55 (1993).

94. Duró, A. & Saiz, E. Distribution and trophic ecology of chaetognaths in the western Mediterranean in relation to an inshore – offshore gradient. *J. Plankton Res.* **22,** 339–361 (2000).

95. Almeda, R., Alcaraz, M., Calbet, A. & Saiz, E. Metabolic rates and carbon budget of early developmental stages of the marine cyclopoid copepod Oithona davisae. *Limnol. Oceanogr.* **56,** 403–414 (2011).

96. Pakhomov, E. a. Salp/krill interactions in the eastern Atlantic sector of the Southern Ocean. *Deep Sea Res. Part II Top. Stud. Oceanogr.* **51,** 2645–2660 (2004).

97. Von Harbou, L. Trophodynamics of Salps in the Atlantic Southern Ocean. *Bereich Biol. Oceanogr.* (2010). at <http://nbn-resolving.de/urn:nbn:de:gbv:46-diss000119205>

98. Giesecke, R., González, H. E. & Bathmann, U. The role of the chaetognath Sagitta gazellae in the vertical carbon flux of the Southern Ocean. *Polar Biol.* **33,** 293–304 (2010).

99. Cosper, T. C. & Reeve, M. R. Digestive efficiency of the chaetognath Sagitta hispida Conant. *J. Exp. Mar. Bio. Ecol.* **17,** 33–38 (1975).
